# Dynamic interfacial trapping of flexural waves in structured plates

**DOI:** 10.1098/rspa.2015.0658

**Published:** 2016-02

**Authors:** S. G. Haslinger, R. V. Craster, A. B. Movchan, N. V. Movchan, I. S. Jones

**Affiliations:** 1 Department of Mathematical Sciences, Mathematical Sciences Building, University of Liverpool, Peach Street, Liverpool L69 7ZL, UK; 2 Department of Mathematics, Imperial College London, London SW7 2AZ, UK; 3 School of Engineering, Liverpool John Moores University, Liverpool L3 3AF, UK

**Keywords:** flexural waves, platonic crystals, discrete Wiener–Hopf method, localization, neutrality

## Abstract

The paper presents new results on the localization and transmission of flexural waves in a structured plate containing a semi-infinite two-dimensional array of rigid pins. In particular, localized waves are identified and studied at the interface boundary between the homogeneous part of the flexural plate and the part occupied by rigid pins. A formal connection has been made with the dispersion properties of flexural Bloch waves in an infinite doubly periodic array of rigid pins. Special attention is given to regimes corresponding to standing waves of different types as well as Dirac-like points that may occur on the dispersion surfaces. A single half-grating problem, hitherto unreported in the literature, is also shown to bring interesting solutions.

## 1. Introduction

The advent of designer materials such as metamaterials, photonic crystals and micro-structured media that are able to generate effects unobtainable by natural media, such as Pendry's flat lens [1], is driving a revolution in materials science. Many of these ideas originate in electromagnetism and optics, but are now percolating into other wave systems such as those of elasticity, acoustics or the idealized Kirchhoff–Love plate equations for flexural waves, with this analogue of photonic crystals being labelled as platonics [2]. Many of the effects from photonics also appear in the flexural wave context, albeit with some changes due to the biharmonic nature of the Kirchhoff–Love equation: ultra-refraction and negative refraction [3–5], Dirac-like cones, Dirac-cone cloaking and related effects [6–8] among others.

The Kirchhoff–Love equations are good approximations within their realm of applicability (for instance with *h*, λ as plate thickness and typical wavelength, respectively, then *h*/λ≪1 is required) and capture much of the essence of the wave physics, hence their emergent popularity. The system is relatively simple so analytic results for infinite periodic structured plates pinned, say, at regular points readily emerge [9], with much of this earlier work reviewed in Mead [10]; more recently multipole methods [11], extending pins to cylinders or high-frequency homogenization approaches [12] to obtain effective continuum equations that encapsulate the microstructure, have emerged. For finite pinned regions of a plate, a Green's function approach [13] leads to rapid numerical solutions, or for an infinite grating one may employ an elegant methodology for exploring Rayleigh–Bloch modes. This includes extensions to stacks of gratings and the trapping and filtering of waves [14–16] which further exemplify this approach. Problems such as these start to pose questions about semi-infinite gratings, or edge states in semi-infinite lattices, and our aim is to generate the relevant exact solutions.

For semi-infinite cracks, and other situations where the change in the boundary condition along a line is of interest, the classical Wiener–Hopf technique [17] for solving integral equations is highly developed and often used for mixed boundary-value problems in continuum mechanics; indeed, the seminal Wiener–Hopf treatise by Noble [18] covers this aspect almost exclusively. However, lesser known [19] and presented only as an exercise in Noble [18], 4.10, pp. 173–174 is its application to continuum discrete problems such as gratings. As such, it has seen notable application to the fracture of discrete lattice systems as reviewed by Slepyan [20], to antenna design [21] and to various semi-infinite grating and lattice scattering problems in acoustics [22,23], where the early work of Hills & Karp [24] (hereafter referred to as HK), motivated by Karp [25], provides a wealth of useful information.

The corresponding exact solutions for the platonic system are not available and, given the current interest in platonics and the versatility of these in, say, asymptotic schemes and the key insight given into the physics and results, we aim to provide these here. Of particular interest are regimes for which one observes the interplay between grating modes and Floquet–Bloch waves in a full doubly periodic infinite structure. In particular, these include frequencies and wavevector components corresponding to stationary points on the dispersion surfaces as well as neighbourhoods of the Dirac-like points. Additionally, we address a fundamental question as to whether a simple, yet surprisingly physically rich, structure such as a half-plane of rigid pins in a plate supports flexural interfacial modes; by which we mean waves that propagate along the interface of the platonic crystal and the homogeneous part of the biharmonic plate.

**Figure 1.**
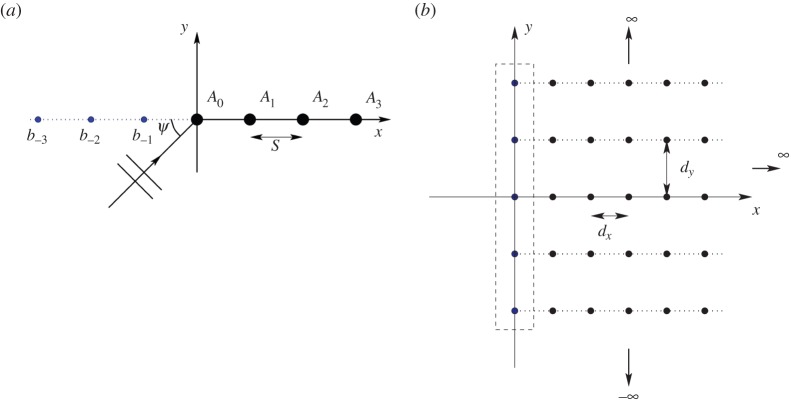
(*a*) A single semi-infinite grating of rigid pins in a biharmonic plate with spacing *s*. The pins are represented by the large black discs. The forces/intensities are represented by the coefficients *A*_*i*_, and displacements, used in the Wiener–Hopf approach, by *b*_*i*_. (*b*) The semi-infinite lattice of pins, consisting of an array of infinite gratings with period *d*_*y*_ in the vertical direction and horizontal spacing *d*_*x*_.

Since the underlying mathematical structure of a semi-infinite linear grating and that of a semi-infinite lattice are very similar, we choose to treat both examples within this paper. For clarity, we shall refer to a grating when we have a single half-line of scatterers (figure 1*a*) and to a lattice when there is a lattice of scatterers in a half-plane (figure 1*b*); throughout this paper, we shall assume that we have point-clamped scatterers although the analysis is readily extended to more complicated conditions holding at a point. In §2, we formulate the problem, drawing upon the discrete Wiener–Hopf technique used by HK for the semi-infinite diffraction grating governed by a Helmholtz operator; much is directly applicable provided we replace the point source Green's function for the two-dimensional Helmholtz operator with that for the biharmonic operator. In some regards, difficulties associated with the Helmholtz Green's function's logarithmic singularity are bypassed for the biharmonic case, where there is no singularity. We turn our attention to the linear grating first, in §2a, and follow that with an analysis of the lattice, in §2b. As noted above this is a parametrically and physically rich problem displaying a surprisingly wide range of behaviours and we outline methodologies for both interpreting and designing specific behaviours; we consider these in §3a,b for the semi-infinite grating and lattice, respectively. Concluding remarks are drawn together in §4.

## 2. Formulation

The methodology primarily adopted here, at least for the exact solution, is that of discrete Wiener–Hopf drawing upon the treatment of the Helmholtz semi-infinite grating by HK, which we briefly review here. To avoid issues with singularities in their problem, HK consider cylinders of finite radius, small compared with wavelength and widely spaced. The diffracted field consists of a cylindrical wave plus a set of plane waves that possess amplitudes and directions of propagation identical to those for an infinite grating, but for the semi-infinite case they do not exist everywhere. Instead the end-effects lead to shadow boundaries defined by lines drawn from the end of the grating to infinity along propagation directions of various plane waves. The cylindrical wave has sharp zeros followed by a maximum for certain directions; these zeros occur in the directions that would be shadow boundaries if the incident wave travels directly into the end of the grating—a class of resonant cases characterized by a diffracted wave that travels parallel to the grating. HK note that, for the wave that travels parallel and out of the grating, the cylindrical wave and all the other plane waves vanish.

Assuming isotropic scatterers HK (see also Linton & Martin [22]) pose the solution in the form

2.1 $$U(r,\theta )=\sum_{n=0}^{\infty }A_{n}H_{0}^{\left(1\right)}(\beta r_{n}),$$

for some coefficients *A*_*n*_ to be determined and $H_{0}^{\left(1\right)}(z)$ being the usual Hankel function of the first kind with wavenumber *β*, and *r*_*n*_ representing the distance between the observation point (*r*,*θ*) and the *n*th scattering element on the *x*-axis; for an infinite array these just differ by a phase factor and the analysis is simpler. The assumption that the grating's elements are small, and therefore scatter isotropically, is not formally required for the pinned biharmonic plate as this is exactly true.

In HK, the spacing between the scatterers is assumed to be large compared with the wavelength, and it must not be an integral or half-integral multiple of the wavelength. These assumptions are required by the mathematical analysis employed. The reason for the limitation on the integral or half-integral multiples of wavelength is to ensure that branch cuts for z=exp(iβs) and z=exp(−iβs) are distinct (see the electronic supplementary material, appendix B, for a discussion of branch cuts for the biharmonic plate), where *s* is the spacing of the pins.

To determine the scattered wave coefficients *A*_*n*_ in (2.1), the boundary conditions generate an infinite system of linear equations solvable using discrete Wiener–Hopf. In the neighbourhood of the *n*th scatterer, the field consists of the incident plane wave, the sum of waves scattered to the *n*th element by the other elements and the wave scattered by the *n*th element itself,

2.2 $$e^{ins\beta \cos\psi }+\sum_{\begin{array}{c} m=0\\ m\neq n \end{array}}^{\infty }A_{m}H_{0}^{\left(1\right)}(\beta |n-m|s)+H_{0}^{\left(1\right)}(\beta a)=0,\;n=0,1,2,\ldots .$$

Here *a* is the radius of the individual elements. The term $H_{0}^{\left(1\right)}(\beta a)$ is treated separately because of the logarithmic singularity arising for the Hankel function at the origin. Although a small radius *a* is assumed by HK, the singularity poses problems as *a*→0 and much of HK involves dealing with this; for the biharmonic case we discuss in this article, the Green's function is finite at the source point rather than diverging logarithmically.

### (a) Semi-infinite grating of rigid pins in a biharmonic plate

The Green's function *G*(*β*,|**r**−**r**′|) for an infinite Kirchhoff–Love plate is given by

2.3 $$G(\beta ,|\text{r}-{\text{r}}^{\prime }|)=\frac{i}{8\beta^{2}}\left[H_{0}^{\left(1\right)}(\beta |\text{r}-{\text{r}}^{\prime }|)+\frac{2i}{\pi }K_{0}(\beta |\text{r}-{\text{r}}^{\prime }|)\right].$$

This function describes an outgoing flexural wave in an isotropic homogeneous thin plate generated by a point source at **r**=**r**′, and it satisfies the equation

2.4 $$\Delta^{2}G(\beta ,|\text{r}-{\text{r}}^{\prime }|)-\beta^{4}G(\beta ,|\text{r}-{\text{r}}^{\prime }|)=\delta (\text{r}-{\text{r}}^{\prime }).$$

As in (2.1) the Green's function (2.3) contains $H_{0}^{\left(1\right)}$, but there is now an additional term containing the modified Bessel function *K*_0_ [26]; this Green's function is bounded at **r**=**r**′.

For a semi-infinite array of rigid pins located at points **r**′_*n*_=(*ns*,0) for spacing *s* and non-negative integer *n*, the amplitude of the scattered field is given by

2.5 $$U(r,\theta )=\sum_{n=0}^{\infty }A_{n}G(\beta ,|\text{r}-{\text{r}}_{n}^{\prime }|),$$

where the coefficients *A*_*n*_ are to be determined. To set up the system of equations, we introduce displacements *b*_*n*_ at (*ns*,0) for all *n*, imposing zero displacements at the pins, *b*_*n*_=0 for *n*≥0; *b*_*n*_ are unknown for *n*<0. For the intensities, we define *A*_*n*_=0 for all *n*<0.

The analogue of (2.2) is

2.6 $$e^{ins\beta \cos\psi }+\sum_{m=0}^{\infty }A_{m}G(\beta ,|n-m|s)=b_{n},\;n\in Z.$$

We now employ the *z*-transform, multiply (2.6) by *z*^*n*^ for the *z* complex, and sum over all *n*,

2.7 $$\sum_{n=-\infty }^{\infty }z^{n}\;e^{ins\beta \cos\psi }+\sum_{n=-\infty }^{\infty }\sum_{m=0}^{\infty }z^{n}A_{m}G(\beta ,|n-m|s)=\sum_{n=-\infty }^{\infty }z^{n}b_{n}.$$

To transform (2.7) into a single functional equation of the Wiener–Hopf type, it is convenient to define *A*(*z*) and a kernel function K(z) as

2.8 $$A(z)=\sum_{m=0}^{\infty }A_{m}z^{m}$$

and

2.9 $$K(z)=\frac{i}{8\beta^{2}}\sum_{j=-\infty }^{\infty }\left[H_{0}^{\left(1\right)}(\beta s|j|)+\frac{2i}{\pi }K_{0}(\beta s|j|)\right]z^{j}.$$

Notably, for K(z), the *j*=0 term is constant, *i*/8*β*^2^, which replaces the awkward $H_{0}^{\left(1\right)}(\beta a)$ term of (2.2). Using these functions, (2.7) becomes

2.10 $$F(z)+A(z)K(z)=B(z),\;\mathrm{where}\;B(z)=\sum_{n=1}^{\infty }b_{-n}z^{-n},$$

and the forcing function, in this case, is

2.11 $$F(z)=\sum_{n=-\infty }^{\infty }{\left(z\;e^{is\beta \cos\psi }\right)}^{n}$$

but could take different forms if the forcing were altered.

Equation (2.10) is the starting point for the Wiener–Hopf technique with key steps being the product factorization of the kernel function K(z) into two factors which are analytic in given but different regions, and factorization of the forcing function. Before proceeding we turn to the semi-infinite lattice as the formulation is almost identical.

### (b) Semi-infinite lattice of rigid pins in a biharmonic plate

We now replace each pin in the semi-infinite grating with an infinite grating in the vertical direction, as illustrated in figure 1*b*. The properties of the infinite grating are well known (e.g. [27]), and the quasi-periodic grating Green's function,

2.12 $$G_{0}^{q}(\beta ,x;\kappa_{y},d_{y})=\frac{i}{8\beta^{2}}\sum_{j=-\infty }^{\infty }\left[H_{0}^{\left(1\right)}(\beta \sqrt{{\left(jd_{y}\right)}^{2}+x^{2}})+\frac{2i}{\pi }K_{0}(\beta \sqrt{{\left(jd_{y}\right)}^{2}+x^{2}})\right]e^{i\kappa_{y}jd_{y}},$$

plays an important role. Here *d*_*y*_ is the vertical period (in §2a we used *s* to denote the period for the single grating) and *κ*_*y*_ is the corresponding Bloch parameter. The gratings are separated by integer multiples of spacing *d*_*x*_ and the semi-infinite lattice is created from columns of infinite gratings (or rows of semi-infinite gratings). The analogue of (2.6) emerges as

2.13 $$e^{ind_{x}\beta \cos\psi }+\sum_{m=0}^{\infty }A_{m}G_{m}^{q}(\beta ,|n-m|d_{x};\kappa_{y},d_{y})=b_{n},\;n\in Z.$$

We repeat the procedure used for the semi-infinite grating with the only difference being that the kernel function is now connected with the doubly quasi-periodic Green's function,

2.14 $$A(z)K(z)=\sum_{n=-\infty }^{\infty }\sum_{m=0}^{\infty }A_{m}G_{n-m}^{q}(\beta ,|n-m|d_{x};\kappa_{y},d_{y})z^{n},$$

when *z*=*e*^*iκ*_*x*_*d*_*x*_^ with $\kappa_{x}=\beta \cos\psi$.

### (c) Wiener–Hopf factorization

The crux of the Wiener–Hopf methodology is to unravel (2.10) using the analyticity properties of the unknown functions *A*(*z*),*B*(*z*) and the known functions K(z), *F*(*z*).

**Figure 2.**
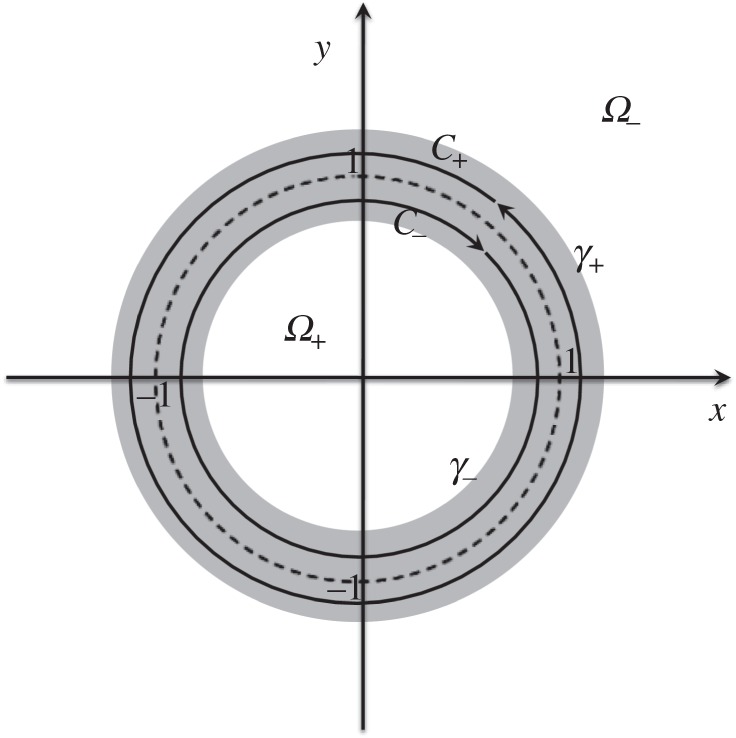
The unit circle (dashed curve), circles *γ*_±_, which define the bounds for the ring of analyticity (shaded region), *Ω*_+_, *Ω*_−_ and contours *C*_+_ and *C*_−_. The integrals on *C*_+_ and *C*_−_ are evaluated counter-clockwise and clockwise, respectively.

Here, a regularization of the kernel function K (2.9) uses complex *β*_*ϵ*_=*β*+*iϵ* instead of the real *β*, where 0<*ϵ*≪1 is a small positive imaginary part. As a result of such a regularization, one creates a ring of analyticity bounded by circles *γ*_±_ (as shown by the shaded region in figure 2), containing the unit circle. Inside this annulus of analyticity, the regularized kernel function does not have any poles. As *ϵ*→0, the circles *γ*_±_ tend to the unit circle, of course.

To proceed we define domains *Ω*_+_ and *Ω*_−_ such that

2.15 $$\Omega_{+}=\{z:|z|\leq c_{+}\}\;\mathrm{and}\;\Omega_{-}=\{z:|z|\geq c_{-}\},$$

where *C*_+_ is a circle of radius *c*_+_=1+*δ*, inside *γ*_+_, and *C*_−_ is a circle of radius *c*_−_=1−*δ*, outside *γ*_−_, for 0<*δ*≪1 (see figure 2 and the electronic supplementary material, appendix A, for more details); the intersection *Ω*_+_∩*Ω*_−_ belongs to an annulus of analyticity in the neighbourhood of the unit circle, and *γ*_±_ are bounds for the circles *C*_±_.

From the definitions of *A*(*z*) (2.8) and *B*(*z*) (2.10), and assuming appropriate decay at infinity, we identify them as + and − functions analytic in *Ω*_+_ and *Ω*_−_, respectively. We then attempt to separate (2.10) into sides entirely analytic in *Ω*_±_; the only way both can be equal, given an overlapping region of analyticity, is for them both to equal the same entire function—thus identifying *A*_+_(*z*) and *B*_−_(*z*). The outcome is

2.16 $$F_{+}(z)+A_{+}(z)K_{+}(z)=\frac{B_{-}(z)}{K_{-}(z)}-F_{-}(z)=0,$$

which requires the product factorization $K(z)=K_{+}(z)K_{-}(z)$ and sum factorization $F(z)=F_{+}(z)+F_{-}(z)$, where $F(z)=F(z)/K_{-}(z)$; the product factorization is highly technical so we give a brief outline here with more details in the electronic supplementary material, appendix A—the sum factorization is straightforward by inspection. The product factorization is

2.17 $$K(z)=\exp\left\{\frac{1}{2\pi i}\int_{C_{+}}\frac{\log K(\varrho )}{\varrho -z}\;d\varrho \right\}\exp\left\{\frac{-1}{2\pi i}\int_{C_{-}}\frac{\log K(\varrho )}{\varrho -z}\;d\varrho \right\}=K_{+}(z)K_{-}(z).$$

We note here that *ϱ* is of the form *e*^*i*(*θ*±*iδ*)^ for *C*_∓_, with *z*=*e*^*iθ*^. It is also assumed that *C*_±_ are chosen so that logK is well defined.

After some algebra, we obtain

2.18 $$\begin{array}{rl} & A_{+}(z)K_{+}(z)+\frac{1}{K_{-}(e^{-i\beta_{\epsilon }s\cos\psi })[1-z\;e^{i\beta_{\epsilon }s\cos\psi }]}\\ & \;=\frac{1}{\left(1-z\;e^{i\beta_{\epsilon }s\cos\psi }\right)}\left[\frac{1}{K_{-}(e^{-i\beta_{\epsilon }s\cos\psi })}-\frac{1}{K_{-}(z)}\right]+\frac{B_{-}(z)}{K_{-}(z)}=0, \end{array}$$

except for different definitions of *A*_+_(*z*), *B*_−_(*z*) and $K_{+}(z)$, $K_{-}(z)$ this mirrors HK. The common entire function is identified, after using Liouville's theorem to extend to the whole complex plane, as zero. Thus

2.19 $$A_{+}(z)=-\frac{F_{+}(z)}{K_{+}(z)}=-\frac{1}{K_{+}(z)K_{-}(e^{-i\beta_{\epsilon }s\cos\psi })[1-z\;e^{i\beta_{\epsilon }s\cos\psi }]},$$

and the *z*-transform for the displacement coefficients *B*_−_(*z*) also follows.

Technical details of the discrete Wiener–Hopf method used here are given in the electronic supplementary material, appendices. The kernel function K(z) is the sum of an infinite series of Hankel and modified Bessel functions, and its factorization is discussed in the electronic supplementary material, appendix A, together with details about the evaluation of the integrals for $K_{+}(z),\;K_{-}(z)$. We also note that the regularization using complex *β*_*ϵ*_, with a small imaginary part, instead of real *β*, ensures that the right-hand side of (2.19) is analytic for all *z* inside *Ω*_+_. The electronic supplementary material, appendix B, provides explanations and formulae required to accelerate the extremely slow convergence of the kernel's series, owing to the highly oscillatory nature of the Hankel function terms and the presence of branch cuts. We also suggest some alternative approaches to accelerate numerical evaluation of the series besides the regularization method primarily implemented here.

## 3. Results

We present the results and illustrative examples for the displacement fields associated with the two types of array: the single semi-infinite pinned platonic grating characterized by spacing *s* in §3a and the two-dimensional lattice defined by *d*_*x*_ and *d*_*y*_ in §3b. We use the discrete Wiener–Hopf method described above and compare the results with those for a truncated semi-infinite array analysed with a method attributable to Foldy [28], which we outline in §3a for a single grating.

### (a) The semi-infinite grating of rigid pins

For a plane wave incident at an angle *ψ* as in figure 1*a*, we determine the coefficients *A*_*k*_ for a truncated grating by solving the algebraic system of linear equations,

3.1 $$\sum_{k=0}^{N}A_{k}G(\beta ,|m-k|s)=-u_{i}(ms),\;m=1,2,\ldots ,N,$$

with *s* being the spacing of the grating's pinned points and *u*_*i*_ the incident wave as defined in equation (2.5). This is the standard Foldy scattering equation [28], and is used among others by Linton & Martin [22] for the Helmholtz problem and Evans & Porter [13] for the biharmonic plate. It is solved for *N* pinned points, and the displacements are plotted using

3.2 $$u(\text{r})=u_{i}(\text{r})+\sum_{k=0}^{N}A_{k}G(\beta ,|\text{r}-(ks,0)|).$$

We compare the results with those obtained from the discrete Wiener–Hopf technique for the semi-infinite grating, which gives us the exact solution. The expressions for $K_{+}(z),K_{-}(z)$ (2.17) are substituted into equation (2.19) to determine *A*_+_(*z*), bearing in mind the contribution arising from the $1-z\;e^{i\beta_{\epsilon }s\cos\psi }$ term in the denominator. Referring to the expansion (2.8), we take the inverse of the *z*-transform to determine the coefficients *A*_*m*_. Multiplying (2.8) through by *z*^−*k*^ and integrating with respect to *z*=*e*^*iθ*^, we obtain

3.3 $$\int_{0}^{2\pi }z^{-k}A_{+}(z)\;d\theta =\int_{0}^{2\pi }\sum_{m=0}^{\infty }A_{m}z^{m}z^{-k}\;d\theta =A_{k}2\pi .$$

The final result for the coefficients is the integral

3.4 $$A_{k}=\frac{1}{2\pi }\int_{0}^{2\pi }\;e^{-ik\theta }A_{+}(e^{i\theta })\;d\;\theta ,$$

for which the singularities and the branch cuts of the electronic supplementary material, appendix B, have to be taken into account. The results compare well with a truncated version (at least 2000 pins) treated with the Foldy scattering approach. In the examples that follow we use both real parts and moduli of the displacement field defined by these complex coefficients, since they give us insight into the propagation tendencies of the scattered waves.

For the far-field behaviour of the coefficients *A*_*k*_, we deduce a relation of the form

3.5 $$A_{k+1}\approx \lambda A_{k},\;|\lambda |\leq 1.$$

The case |λ|=1 denotes the propagating Bloch wave and for |λ|<1 we observe localization linked with the exponential decay of the coefficients. The ratio λ is determined by

3.6 $$\lambda =\lim_{k\to \infty }\frac{\int_{C}A_{+}(z)z^{-(k+1)}\;dz}{\int_{C}A_{+}(z)z^{-k}\;dz},$$

where the kernel in its present form is evaluated numerically.

#### (i) Resonant cases

We consider some of the frequency regimes mentioned by Evans & Porter [13] for a finite array of pinned points and an infinite grating, as well as those referred to by HK for a semi-infinite grating, including the case they define as a resonance. This is where the incident wave travels directly into the end of the grating and then the diffracted wave travels parallel to the grating, either into (inward resonance) or away from (outward resonance) the grating.

Spectral orders of diffraction *ϕ*_*p*_ are defined according to the equation (see for instance [29])

3.7 $$\cos\phi_{p}(\psi )=\cos\psi +\frac{2\pi p}{s\beta }\;p\in Z,$$

with only a finite number of the *ϕ*_*p*_(*ψ*) being real and representing propagating waves. The remaining orders are complex and represent evanescent waves. According to HK, the resonant cases arise when one of the spectral directions is zero (inward resonance) or *π*, the case of outward resonance. Thus, resonances coincide with additional diffraction orders becoming propagating. For outward resonance

3.8 sβcosψ+2πp=−sβ,for some p=0,±1,±2,…,

thus for *ψ*=0, *sβ*=−*πp* and outward resonances occur for frequencies corresponding to *β* being a multiple of *π*.

**Figure 3.**
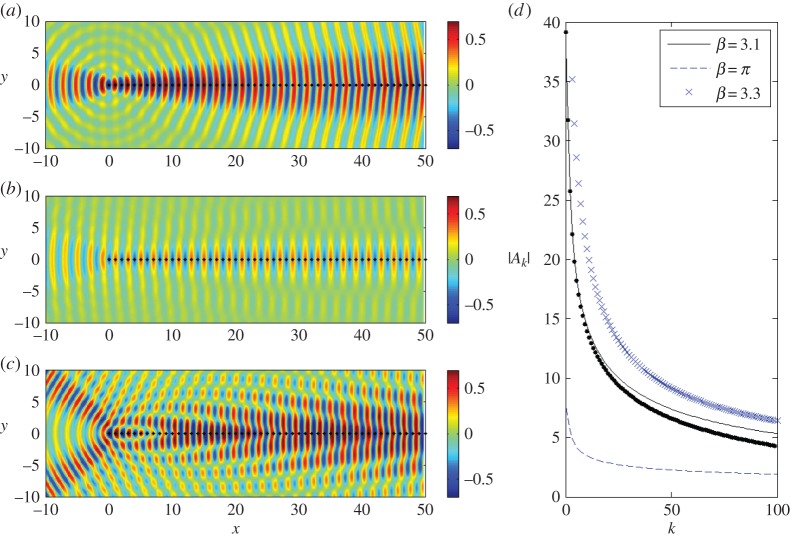
Plane wave incident on a semi-infinite grating with spacing *s*=1.0 at *ψ*=0. (*a*–*c*) Real part of the scattered displacement *u*−*u*_*i*_, for *β*=3.1,*π*,3.3. (*d*) Comparison of the moduli of the coefficients |*A*_*k*_| using the Wiener–Hopf (dots for *β*=3.1) and Foldy methods (shown for all). (Online version in colour.)

We observe some evidence of this effect for the case *p*=−1 in figure 3*a*–*c*, where we plot the scattered displacement fields for values of *β*=3.1,*π*,3.3 for angle of incidence *ψ*=0 and period *s*=1.0. In figure 3, we use both Foldy, truncated to 2000 pins, and the results from Wiener–Hopf, and they are visually indistinguishable for the first several gratings, as shown in figure 3*d*, which compares the moduli of the coefficients |*A*_*k*_| in both cases for *β*=3.1 (i.e. the solid line and dots for Foldy and Wiener–Hopf, respectively). The high reflected energy for *β*=3.1 shown in figure 3*a* is illustrative of the outward resonance described by HK for the resonant value *β*=*π*.

We contrast this with *β*=3.3 in figure 3*c* for which an additional diffraction order *p*=−1 has become propagating. For diffraction orders *p*<0 to pass off, βs(1+cosψ)=−2πp. Thus for *ψ*=0, the order *p*=−1 becomes propagating along with *p*=0 for the resonant value *β*=*π*, and the presence of two distinct propagating orders is clearly illustrated for *β*=3.3. The two examples either side of *β*=*π* show strong evidence of the circular wave that is typical for the end-effects of a semi-infinite scatterer.

The resonant frequency case of *β*=*π* is shown in figure 3*b* and there are relatively lower amplitudes of displacement (with a maximum of approx. 0.3) with most of the scattering occurring along the grating, contrasting with the effects illustrated in figure 3*a*,*c*. The localization and reduction of the maximum amplitudes to 0.3 is consistent with HK's observation that, for outward resonance, the cylindrical wave and all but one of the plane waves vanish. Indeed, the real part of the total displacement field (not shown) closely resembles that of the incident wave, except for the region surrounding the grating itself. Comparing the coefficients *A*_*k*_, as we do in figure 3*d*, further emphasizes the difference between this resonant case, for which |*A*_*k*_| rapidly saturates to a low value, and those for *β*=3.1,3.3, which take much longer to saturate and then do so to a larger value.

#### (ii) Shadow boundaries

HK refer to non-resonant cases where the diffracted field for a Helmholtz-governed semi-infinite grating consists of a set of plane waves and a cylindrical wave. The plane waves are consistent with those arising for the infinite grating, but do not exist everywhere. We obtain similar results for the biharmonic case, for which the propagating waves are due to the Hankel functions arising from the Helmholtz part of the Green's function in equation (2.3). The coefficients for the scattered field are defined by equations (2.19) and (3.4), leading to the expression

3.9 $$A_{k}=-\frac{1}{2\pi K_{-}(e^{-i\beta s\cos\psi })}\int_{0}^{2\pi }\frac{e^{-ik\theta }\;d\theta }{K_{+}(e^{i\theta })[1-e^{i\theta }\;e^{i\beta s\cos\psi }]}.$$

Furthermore, the representation (2.5) for the displacement leads to the following approximate expression for large values of |**r**−**r**′_*n*_|:

$$u(r,\phi )≃\frac{i}{8\beta^{2}}\sum_{n=0}^{\infty }A_{n}H_{0}(\beta |\text{r}-{\text{r}}_{n}^{\prime }|).$$

HK have proved in their paper that the shadow boundaries correspond to singularities of the *z*-transform *A*_+_(*z*) (2.19), when *z* is a point on the unit circle defined in the form $z={\text{e}}^{-i\beta s\cos\phi_{p}}$. We note that the regularized kernel K(z) does not have roots and poles on the unit circle, and hence the shadow boundaries are defined by equation (3.7) and coincide with those discussed by HK in [24].

**Figure 4.**
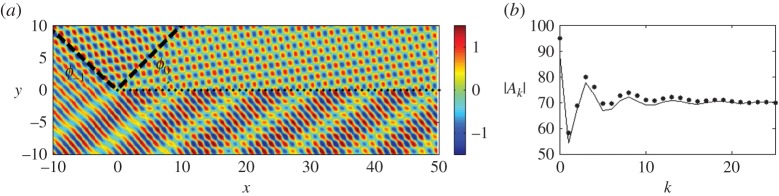
The scattering of a plane wave *ψ*=*π*/4 by a grating with *s*=1.0, for *β*=4.0. (*a*) Real part of the displacement field *u*. The shadow boundaries for *ϕ*_0_ and *ϕ*_−1_ are marked using dashed black lines. (*b*) Comparison of the Wiener–Hopf (dots) and Foldy (solid line) coefficients |*A*_*k*_|. (Online version in colour.)

The lines *θ*=*ϕ*_*p*_(*ψ*) act as shadow boundaries and this is illustrated in figure 4 where *β*=4.0, *s*=1.0 and *ψ*=*π*/4. For *ψ*=*π*/4, the diffraction order *p*=−1 becomes propagating for $\beta =2\pi /(1+1/\sqrt{2})\approx 3.6806$, so for *β*=4.0 both *p*=0 and −1 are propagating. We observe the two shadow boundaries for *ϕ*_0_(*π*/4)=*π*/4 and $\phi_{-1}(\pi /4)=\arccos(\sqrt{2}-\pi )/2$, and the field consisting of two propagating diffracted waves, as well as the reflected wave. Figure 4*b* complements figure 4*a* by showing a comparison of the moduli of the coefficients |*A*_*k*_| with the Foldy method represented by the solid line, the Wiener–Hopf by the dots; for oblique incidence, the two methods match well and for the Wiener–Hopf we set *ϵ*=0.005, *δ*=0.0025 and take 1200 intervals for the trapezoidal integration (see the electronic supplementary material, appendices A and B). Increasing the number of intervals leads to closer convergence to the Foldy coefficients.

#### (iii) Reflection and transmission

**Figure 5.**
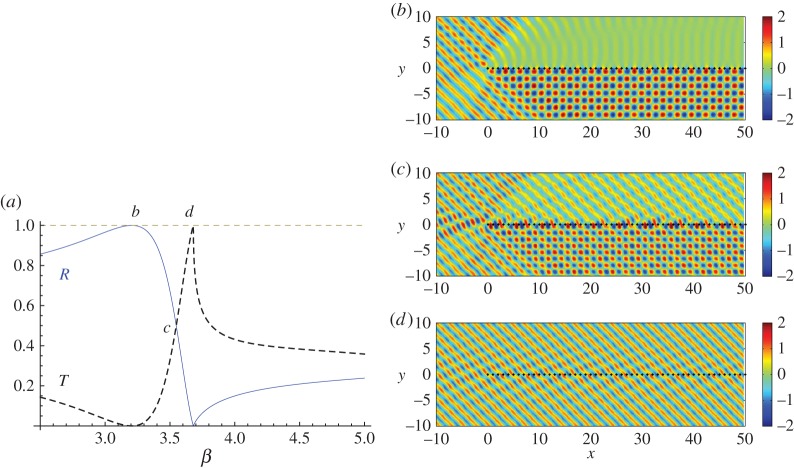
Plane wave incident on a semi-infinite grating with spacing *s*=1.0 at an angle *ψ*=*π*/4. (*a*) The normalized reflected, *R*, (solid curve) and transmitted, *T*, (dashed) energy versus *β* for scattering by an infinite pinned grating. The real part of the displacement field for the semi-infinite grating is shown for (*b*) *β*=3.2, (*c*) *β*=3.55 and (*d*) *β*=3.68. Fields for parts (*b*–*d*) are plotted using Wiener–Hopf coefficients. (Online version in colour.)

Frequency regimes associated with total reflection and total transmission by an infinite grating, as identified by Evans & Porter [13] and Movchan *et al.* [14], are interesting cases. For an *infinite* pinned grating (period *s*=1.0, *ψ*=*π*/4), the normalized reflected and transmitted energies are plotted against *β* in figure 5*a*. Three important values of *β* = 3.2; 3.55; 3.68, which are total reflection, equipartition of energy and total transmission, respectively, are labelled *b*–*d* and we show the real part of the total displacement field for the corresponding *semi-infinite* gratings in figure 5*b*–*d*.

Figure 5*a* shows that the maximum reflected energy (solid curve) for the zeroth propagating order arises for *β*=3.2. The Wood anomaly at *β*=3.6806 signifies the passing off of the order *p*=−1, explaining the multiple orders illustrated in figure 4 for *β*=4.0. The real part of the total displacement field for the semi-infinite grating for the same parameter values *ψ*=*π*/4 and *β*=3.2 shows strong reflection to the right of the vertex, consistent with that observed for the full grating for the single propagating plane wave. This contrasts sharply with the results observed for the frequency associated with *β*=4.0 in figure 4. Note that there is only one shadow boundary in figure 5*b* (for the shadow region behind the grating), whereas figure 4*a* shows evidence of two distinct shadow lines.

In figure 5*c*, we highlight *β*=3.55, which supports a mixture of reflected and transmitted energy for both the infinite and semi-infinite gratings; the dashed line in figure 5*a* represents the transmitted energy. The real part of the displacement field in figure 5*c* is consistent with the information provided by the energy diagrams for the corresponding infinite grating; both reflection and transmission are visible, with the shadow region above the grating now admitting transmittance. This transmission effect dilutes the reflection of figure 5*b*, and total reflectance is converted to total transmission by adjusting the *β* parameter appropriately. This is illustrated in figure 5*a* where *β*=3.68 leads to full transmission for the infinite grating, and produces a similar effect for the semi-infinite grating whose total displacement field is shown in figure 5*d*. This transmission resonance is an example of the outward resonant effect for oblique incidence. The scattered field (not shown) is reminiscent of figure 3*b*; evidence of the outward-travelling wave close to the vertex is visible in figure 5*c* for *β*=3.55.

### (b) Semi-infinite lattice of rigid pins

For an incident plane wave characterized by *ψ* as in figure 1*a*, we determine the coefficients *A*_*k*_ for a truncated half-plane by solving the algebraic system of linear equations (3.1) but replacing each pin with a grating in the vertical direction. We compare results with those obtained using discrete Wiener–Hopf. We clarify that, whenever the figure caption gives a finite number of gratings, the computation has been performed using Foldy's method.

#### (i) Zeros of the kernel function

Recall from equations (2.12), (2.14) that, for $z=\exp\{i\kappa_{x}d_{x}\}$ lying on the unit circle, the function K(z) is precisely the doubly quasi-periodic Green's function. Therefore, its zeros represent points on the dispersion surfaces for Bloch waves in the infinite doubly periodic structure. This direct connection between the kernel and the doubly periodic medium enables us to analyse wave phenomena using dispersion surfaces, band diagrams and slowness contours.

We determine the coefficients *A*_*k*_ and *b*_*k*_ using the discrete Wiener–Hopf method. A good approximation to these results is obtained by applying Foldy's method to a large enough array. For systems of at least 2000 gratings, we demonstrate several interesting wave phenomena. McPhedran *et al.* [8] highlight the potential for varying the aspect ratio of rectangular lattices of pins, linking Dirac-like points with parabolic profiles in their neighbourhood. The characteristic of this parabolic profile determines the direction of propagation of localized waves. We consider first a semi-infinite rectangular lattice with aspect ratio $\eta =d_{y}/d_{x}=\sqrt{2}$. The band diagram for the doubly periodic rectangular array is shown in fig. 7 of McPhedran *et al.* [8]. Here we represent the dispersion surfaces with isofrequency contour diagrams.

**Figure 6.**
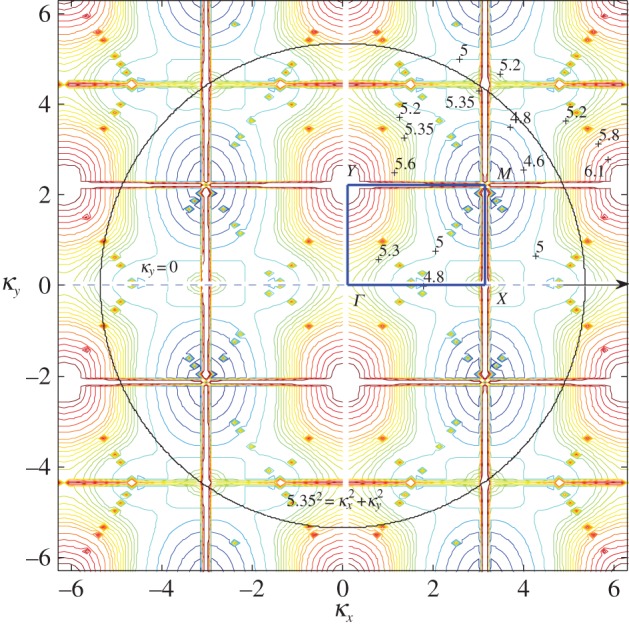
Isofrequency contours (a selection of *β* values are labelled) for the third dispersion surface of the rectangular lattice with *d*_*x*_=1, $d_{y}=\sqrt{2}$. The boundary of the irreducible Brillouin zone *ΓXMY* is marked by the solid rectangle, with $M=(\pi ,\pi /\sqrt{2})$. (Online version in colour.)

For our initial investigations, we focus on the first three dispersion surfaces. In figure 6, we illustrate the third surface for the rectangular lattice with *d*_*x*_=1.0 and $d_{y}=\sqrt{2}$ with the boundary of the irreducible Brillouin zone labelled by *ΓXMY* , where $M=(\pi ,\pi /\sqrt{2})$. The rectangular grid *k*_*x*_=±*πn*/*d*_*x*_, *k*_*y*_=±*πn*/*d*_*y*_ is due to singularities in the dispersion equation. The distortions near the grid should be disregarded. We observe a parabolic cylinder profile along *ΓX* with an inflection at *Γ* where the contours change direction for *β*≈5.365 and a Dirac-like point for *β*≈5.45 at *X*.

The notion of ‘light surfaces’ and ‘light lines’ is well known in the modelling of Bloch waves. The term originally came from electromagnetism, but is now well used in problems of acoustics and elasticity. In particular, in §4 of their article, McPhedran *et al.* [8] give an accurate account of ‘light cones’ for flexural waves in Kirchhoff plates. In this context, the ‘light cones’ are defined as surfaces in the space (*k*_*x*_,*k*_*y*_,*β*) given by the equations

3.10 $$\begin{array}{rl} \eta^{2}{\left(n_{1}+\frac{\kappa_{x}d_{x}}{2\pi }\right)}^{2}+{\left(m_{1}+\frac{\kappa_{y}d_{y}}{2\pi }\right)}^{2} & ={\left(\frac{\beta d_{y}}{2\pi }\right)}^{2}\\ \text{and}\;\;\eta^{2}{\left(n_{2}+\frac{\kappa_{x}d_{x}}{2\pi }\right)}^{2}+{\left(m_{2}+\frac{\kappa_{y}d_{y}}{2\pi }\right)}^{2} & ={\left(\frac{\beta d_{y}}{2\pi }\right)}^{2}. \end{array}\}$$

We also note that the concentration of points in figure 6 tending towards the straight lines

$$\kappa_{y}\pm \left(\frac{\pi +2\pi d_{y}}{2\pi d_{y}}\right)\kappa_{x}=\pm \frac{\pi }{d_{y}}$$

and periodic shifts by *π*/*d*_*y*_ are projections (onto the *κ*_*x*_,*κ*_*y*_-plane) of intersections of two light cones, which are given by (3.10).

The remaining surfaces are presented together with displacement fields in the examples that follow. Both *β*=3.1538 for the first surface and *β*=4.40 for the second surface are mentioned by McPhedran *et al.* [8]. This immediately gives us some frequency regimes to investigate for finding wave phenomena including neutrality and interfacial waves. Recall from the Introduction that we define interfacial waves to be exponentially localized within the structure of pins and propagating parallel to the interface of the platonic crystal and the homogeneous part of the biharmonic plate. We also demonstrate blocking and resonant behaviour.

#### (ii) Wavevector diagrams

Zengerle [30] demonstrated an elegant strategy using wavevector diagrams to investigate wave phenomena in planar waveguides; the technique was also outlined by Joannopoulos *et al.* [31] for photonic crystals in their ch. 10. Zengerle [30] was able to illustrate negative refraction, focusing and interference effects, having predicted them with careful analysis of the wavevector diagrams. We adopt an analogous approach for this platonic crystal system.

The underlying principle is a corollary of Bloch's theorem; in a linear system with discrete translational symmetry the Bloch wavevector **k**=(*κ*_*x*_,*κ*_*y*_) is conserved as waves propagate, up to the addition of reciprocal lattice vectors. Since there is only translational symmetry along directions parallel to the interface, only the wavevector parallel to the interface, **k**_∥_, is conserved. In our case, this is precisely the direction *κ*_*y*_. Thus for any incident plane wave defined by *β* and **k**=(*κ*_*x*_,*κ*_*y*_), any reflected or refracted (transmitted) wave must also possess the same frequency *β*^2^=*ω* and wavevector (*κ*′_*x*_,*κ*_*y*_+2*πl*/*d*_*y*_) for any integer *l* and some *κ*′_*x*_. Therefore, wavevector diagrams may be used to analyse the reflection and refraction of waves within the pinned system.

These isofrequency diagrams (also called slowness contour plots) consist of dispersion curves for constant *β* (characterizing the platonic crystal) and the contour for the ambient medium (the homogeneous biharmonic plate) on the same *κ*_*x*_, *κ*_*y*_ diagram (figure 6). The incident wavevector (whose group velocity direction is characterized by *ψ*) is appended to the ambient medium's contour (the circle $\beta^{2}=5.35^{2}=\kappa_{x}^{2}+\kappa_{y}^{2}$ in figure 6), and a dashed line perpendicular to the interface of the platonic crystal and the homogeneous part of the biharmonic plate (*κ*_*x*_=0 or *ΓY* direction here) is drawn through this point. The dashed line *κ*_*y*_=0 and *ψ*=0 has been added to figure 6. The places where this dashed line intersects the platonic crystal contours determine the refracted waves and their directions, such that their group velocity is perpendicular to the *β* contours, and points in the direction of increasing *β*.

Additional information relating to the coefficients *A*_*k*_ is obtained from the Wiener–Hopf method. Exponential decay of form (3.5) with λ<1 would indicate the possibility of interfacial waveguide modes, with verification supported by the prediction of the refracted waves' directions using wavevector diagrams. We illustrate the technique with an introductory example demonstrating a localized mode along the edge of the crystal for the first dispersion surface, as well as reflection and transmission which are predicted from the isofrequency diagram.

#### (iii) Reflection, transmission and interfacial waves for the first dispersion surface

**Figure 7.**
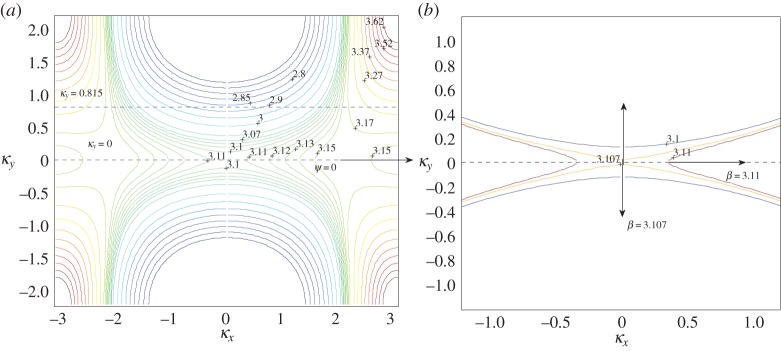
(*a*) Isofrequency contours *β*= constant for the first dispersion surface for the rectangular lattice with *d*_*x*_=1.0, $d_{y}=\sqrt{2}$. (*b*) Isofrequency contours *β*=3.10, 3.107, 3.11. (Online version in colour.)

In figure 7*a*, we show a collection of isofrequency contours for constant *β* for the first dispersion surface for the rectangular lattice with aspect ratio $\eta =d_{y}/d_{x}=\sqrt{2}$. This range of frequencies is notable for its flat bands and the parabolic profile mentioned by McPhedran *et al.* [8] at *β*=3.1538 in the vicinity of a Dirac-like point. For normal incidence *ψ*=0, indicated by the direction of the arrow in figure 7*a*, the dashed line intersecting the ambient medium's contour and normal to the interface (*κ*_*x*_=0) corresponds to *κ*_*y*_=0, as shown in figure 7*a*. We seek the intersections of this line with the isofrequency contour for a specific choice of *β*. Since the direction of the group velocity of the refracted waves is perpendicular to these *β*=*const*. contours, for an interfacial mode we would require the contour to be tangent to the line *κ*_*y*_=0.

#### (iv) Interfacial waves

**Figure 8.**
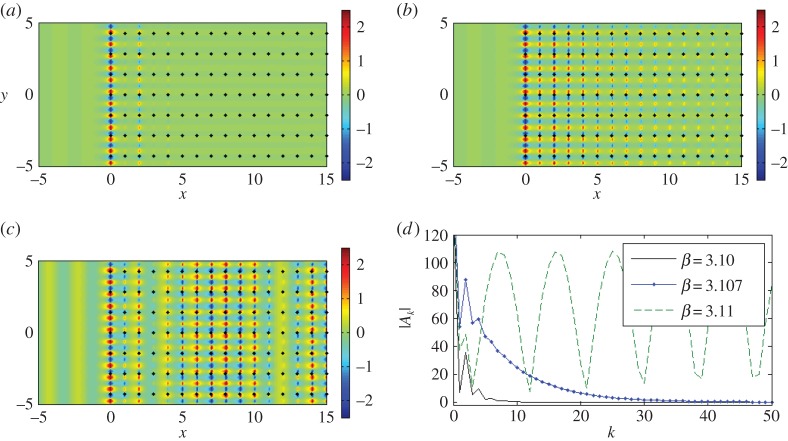
A plane wave is incident at *ψ*=0 on a lattice of 2000 gratings with *d*_*x*_=1.0, $d_{y}=\sqrt{2}$. (*a*–*c*) Real part of the total displacement field for *β*=3.10, *β*=3.107, *β*=3.11. (*d*) Comparison of the moduli of coefficients |*A*_*k*_|. Computations performed using the Foldy method. (Online version in colour.)

It is clear from figure 7*a* that the contours tend towards tangency as *β* increases to *β*≈3.1 but then their behaviour in the vicinity of *β*=*π* is less predictable. This is connected both with the resonant case *β*=*π* for the single semi-infinite grating (see §3a(i)) and with the parabolic profiles associated with Dirac-like points to which McPhedran *et al.* [8] alluded for *β*≈3.1538. This observation is supported by the discontinuous nature of the contours for 3.11≤*β*≤3.15 when they intersect the line *κ*_*y*_=0 in figure 7*a*, and in particular the two distinct contour curves labelled by *β*=3.15 on the right. The contours change direction at *Γ*=(0,0) for 3.10<*β*<3.11, as shown clearly in figure 7*b*; this corresponds to a point of inflection on the band diagram, a case that exhibits interesting wave phenomena that we illustrate in figure 8 for the semi-infinite lattice.

In figure 7*b*, we show isofrequency contours for *β*=3.10, 3.107 and 3.11 to emphasize the transition of the contours through this narrow frequency window. The curve for *β*=3.10 is consistent with preceding values of *β* but it does not touch the *κ*_*y*_=0 line, whereas *β*=3.107 is extremely close to touching, with the origin of a point of discontinuity clearly observable. This point of inflection for some 3.107<*β*<3.11 is the limit as the contours tend to *κ*_*y*_=0 and occurs at *Γ*. For this value of *β*, the refracted wave would travel along the interface, but there would be no preferential direction of propagation since the upper contour would indicate a ‘downward’ direction (increasing *β*), whereas the lower contour would indicate the opposite direction. This suggests the presence of a standing wave, localized within the first few gratings of the half-plane of pins.

Figure 8*a*–*d* gives an interesting illustration of the scattering patterns near the edge of the stop band for a doubly periodic ‘platonic crystal’. The parameters of the lattice and the frequencies are chosen according to figure 7, and correspond to a ‘parabolic regime’, which supports a unidirectional propagation. This choice has been refined, and it is not just an arbitrary example of stop-pass-band excitation. In particular, figure 8*a* shows a very sharp localization, evidence for which is provided by the accompanying graph of the coefficients in figure 8*d*. It is also demonstrated that a small perturbation of the frequency, as in figure 8*c*, leads to a propagating oscillatory pattern inside the structured half-plane.

We see evidence of some localization and some propagation when we plot the real part of the displacement field for *β*=3.107 in figure 8*b*, with the predicted preferential direction of propagation indicated in figure 7*b*. The moduli of the coefficients |*A*_*k*_| are plotted versus the *x*-position of the grating in figure 8*d*, for the three values of *β* featured in figure 7*b*. Both *β*=3.10 and *β*=3.107 show exponential decay of the coefficients, suggesting localization of waves, whereas the steady oscillation of the coefficients for *β*=3.11 predicts transmission of the waves through the system. Figure 8*b* clearly illustrates the localization of waves within the first five gratings, and a clearer example of an interfacial wave is illustrated in figure 8*a* for *β*=3.10, although comparison with lower values of *β* shows similar results, but with more striking reflection action.

#### (v) Transmission

Figure 7*b* predicts that, for *β*=3.11, the preferred direction of propagation of the refracted waves is normal to that for *β*=3.107, in the form of transmission through the system. Note that the opposite direction (*θ*=*π* rather than 0) is ruled out since any intersections corresponding to the group velocity directed towards the interface from the crystal would violate the boundary conditions. The coefficients' behaviour in figure 8*d* also supports the hypothesis of propagating waves. This is demonstrated in figure 8*c*, where we observe transmission as the wave propagates through the system without any change in direction, with the period of the wave's envelope function consistent with the period for the coefficients in figure 8*d*.

#### (vi) Coupling with finite grating stacks

The spikes in figure 8*d* for *β*=3.10 and *β*=3.107 seem to indicate resonant interaction with the first few gratings of the semi-infinite array, as does the localization evident in figure 8*a*,*b*. There is a connection with the finite grating stacks analysed by Haslinger *et al.* [27]; resonances associated with the Bloch modes for the finite systems are linked to the neighbourhood of the Dirac-like point illustrated in figure 7*a*,*b*.

For the pinned waveguide consisting of an odd number of gratings of period *d*_*y*_, a quasi-periodic Green's function (2.12) is used to derive the dispersion equation for Bloch modes within the system. At each pin, the boundary conditions are *u*=0. Therefore, for a system of aligned gratings with spacing *d*_*x*_,

3.11 $$u({\text{a}}^{m})=\sum_{j=-M}^{M}S_{j}G_{j}^{q}(\beta ,md_{x};\kappa_{y},d_{y})=0;\;{\text{a}}^{m}=(md_{x},0)\;\text{for }m\in [-M,M],$$

where *u* is the displacement, *S*_*j*_ are coefficients to be determined for each Green's function $G_{j}^{q}$ and 2*M*+1 is the number of gratings in the finite stack. This is equivalent to the matrix equation

3.12 GS=0,

where **S** is the column vector of coefficients *S*_*j*_ and the Green's function matrix **G** is complex and symmetric Toeplitz. The accompanying dispersion equation is

3.13 Det(G)=0,

the solutions of which characterize the system's Bloch modes.

**Figure 9.**
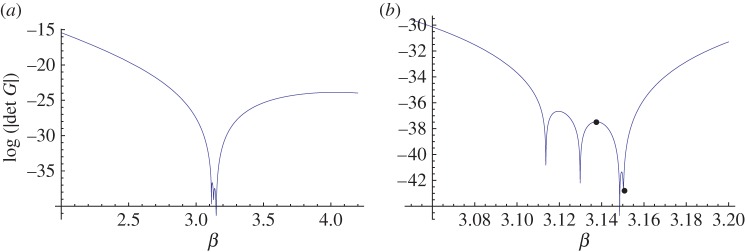
(*a*) Solutions of the eigenvalue problem for a system of five gratings with period $d_{y}=\sqrt{2}$ and separation *d*_*x*_=1.0. (*b*) Close-up of the frequency window for Bloch mode frequencies; local maximum *β*=3.1375 and root *β*=3.151 are marked. (Online version in colour.)

Figure 8*b* features localized waves within the first five gratings so we solve the eigenvalue problem for a system of five gratings with period $d_{y}=\sqrt{2}$ and separation *d*_*x*_=1.0 in figure 9*a*. The frequencies for the 5-grating system's Bloch modes coincide with the frequency window for the Dirac-like point of the doubly periodic rectangular array. Similar results have been obtained for all 2*M*+1-grating stacks with *M*≤6, with localized modes always confined to the range 3.10<*β*<3.17. This is consistent with figure 7*a*,*b*, where the contours are well behaved for *β*≤3.10 and ≥3.17 but exhibit discontinuities in the neighbourhood of the Dirac-like point.

**Figure 10.**
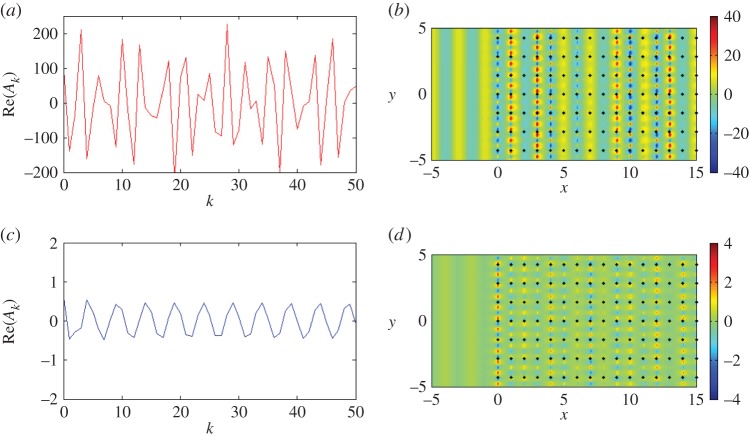
A plane wave is incident at *ψ*=0 on a lattice of 2000 gratings with *d*_*x*_=1.0, $d_{y}=\sqrt{2}$. (*a*) Real part of the coefficients *A*_*k*_ for *β*=3.15138. (*b*) Real part of the total displacement field for *β*=3.15138. (*c*) Real part of the coefficients *A*_*k*_ for *β*=3.1375. (*d*) Real part of the total displacement field for *β*=3.1375. (Online version in colour.)

It has been shown that a finite system's resonant Bloch modes may be coupled to appropriately chosen incident plane waves to generate transmission action [27]. We observe the same phenomenon for the half-plane in figure 8*c* as well as evidence of similar localization for *β*=3.10, *β*=3.107 in figure 8*a*,*b*. Figure 9*b* adds further insight when we select two specific values of *β* marked on the diagram: the local maximum for *β*≈3.1375 and the root *β*=3.151, which is close to the Dirac-like point value *β*=3.1538. In figure 10*a*, the real parts of the coefficients *A*_*k*_ for *β*=3.1538 are of order 10^2^ (the accompanying total displacement field is shown in figure 10*b*), whereas for *β*=3.1375 in figure 10*c*,*d* they are <1. One should take note of the scaling bars when comparing the total displacement fields in figure 10*b*,*d*. Both *β* values in the vicinity of the Dirac-like point support propagation, but the intensities of the transmitted waves are linked to the turning points and roots for the finite grating stack structures, illustrated in figure 9.

This coupling can be switched off by altering the period and/or separation of the gratings or the incoming angle of incidence. It should be noted that these examples for the lowest frequency band involve the zeroth propagating order only. Higher frequencies introduce additional diffraction orders and their coupling facilitates more exotic wave phenomena including negative refraction, similar to those recorded by Zengerle [30] for optical waveguides.

#### (vii) Neutrality in the vicinity of Dirac-like cones

We now consider neutrality effects that arise for parabolic profiles in the vicinity of Dirac-like cones. It is well known that, close to Dirac points, waves may propagate as in free space, unaffected by any interaction with the microstructure within the crystal medium. These directions of neutral propagation around the Dirac point engender cloaking properties within the crystal, meaning that there is potential for ‘hiding’ objects within the appropriate frequency regime. This property of neutrality for platonic crystals was mentioned by McPhedran *et al.* [2] and was explained in a simple way in terms of the singular directions of the Green's functions for the biharmonic equation. More recently, Farhat *et al.* [32] have discussed neutrality when studying the scattering cancellation technique for flexural waves in elastically uniform thin plates.

**Figure 11.**
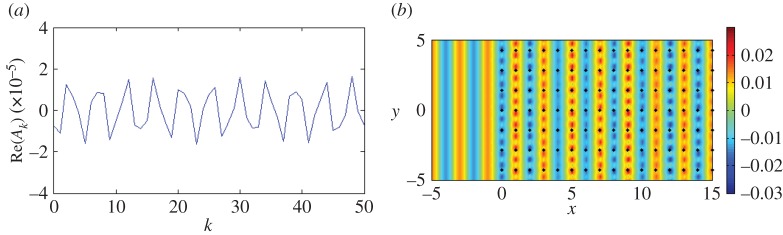
A plane wave is incident at *ψ*=0 on a lattice of 4000 gratings with *d*_*x*_=1.0, $d_{y}=\sqrt{2}$ for *β*=*π*. (*a*) Real parts of the coefficients *A*_*k*_. (*b*) Real part of the scattered field. (Online version in colour.)

The first dispersion surface for the rectangular lattice with *d*_*x*_=1.0, $d_{y}=\sqrt{2}$ in figure 7 possesses such a parabolic profile near *β*=*π*. It is the horizontal spacing of *d*_*x*_=1.0 that governs the special behaviour for normal incidence *ψ*=0 at *β*=*π*, illustrated in figure 11 (see equation (3.8)). For the vertical period $d_{y}=\sqrt{2}$, we observe neutral propagation of the incident plane wave, similar to that observed for the single grating in figure 3*b*, and we also see this for *ψ*=0 for the square lattice. Since the total displacement and incident fields are visually indistinguishable, we provide the real parts of the coefficients *A*_*k*_ in figure 11*a* and the real part of the scattered field in figure 11*b*. It is clear that for this choice of *β*=*π* the wave does not ‘see’ this specific lattice with *d*_*x*_=1.0, $d_{y}=\sqrt{2}$.

#### (viii) Neutrality for oblique incidence

**Figure 12.**
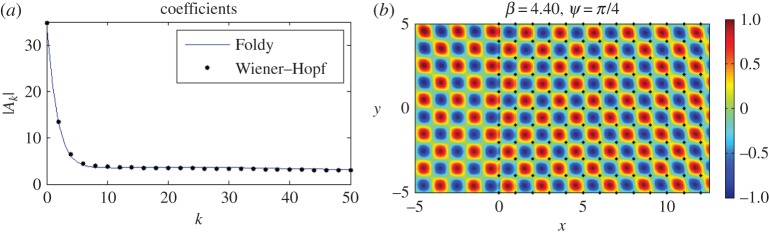
A plane wave is incident at *ψ*=*π*/4 on a lattice of 2000 gratings with *d*_*x*_=1.0, *d*_*y*_=1.0 for *β*=4.40, *κ*_*y*_=3.1113. (*a*) Modulus of the coefficients |*A*_*k*_| for both Wiener–Hopf (dots) with *δ*=0.005 and Foldy (solid line). The first 50 are shown. (*b*) Real part of the total displacement field. (Online version in colour.)

For illustrative purposes, we consider a square lattice of pins with aspect ratio *η*=*d*_*y*_/*d*_*x*_=1 before moving back to the rectangular lattice with $\eta =\sqrt{2}$. In figure 12, a plane wave is incident at *ψ*=*π*/4 with the Bloch parameter in the *y*-direction set to $\kappa_{y}=\beta \sin(\psi )=3.1113$.

The real part of the total displacement field is shown in figure 12*b*, and it appears that the direction of the incident field is virtually unchanged as it passes through the array of pins. Another notable feature is that the stripes of the incident plane wave are replaced by spots of positive and negative intensity; this pattern is due to the individual scatterers and is linked to the neutrality arising from the Dirac-like point. Figure 12*a* illustrates the moduli of the scattering coefficients |*A*_*k*_| obtained using both the Wiener–Hopf (dots) and Foldy (solid line) methods. The agreement is very good, virtually indistinguishable for the first 50 gratings, and for both approaches the coefficients decay to a non-zero constant supporting the neutral propagation that we observe in figure 12*b*. One other interesting feature is that this propagation is not the only wave action; the appearance of ‘spots’ to the left of the crystal indicates that some reflection action is also present.

**Figure 13.**
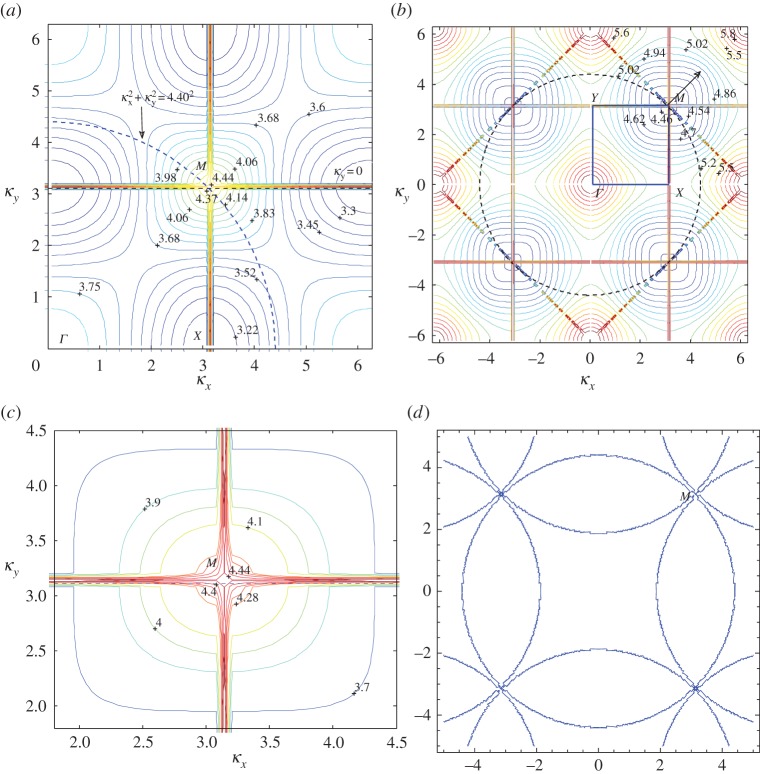
(*a*) Isofrequency contours for the first surface for the square lattice with *d*_*x*_=1.0, *d*_*y*_=1.0. The ambient medium's contour for *β*=4.40 is the dashed circle $\beta^{2}=\kappa_{x}^{2}+\kappa_{y}^{2}$. (*b*) Isofrequency curves for the second surface. The dashed line *κ*_*y*_=3.1113 has been drawn to correspond with Bloch waves in the vertical direction for *β*=4.40, *ψ*=*π*/4. The boundary of the irreducible Brillouin zone *ΓXMY* is also shown. (*c*) Close-up of isofrequency contours around *β*=4.40. (*d*) Star-like features of the dispersion curves for *β*=4.40 at *M* and collection of light circles for the square lattice (note the multiple intersections of the light lines at Dirac-like points). (Online version in colour.)

In figure 13, we use isofrequency diagrams to aid our interpretation of this neutrality effect. In figure 13*a*, we show the isofrequency contours for the first surface for the square lattice, and in figure 13*b* the second surface since *β*=4.40 is in the vicinity of Dirac-like cones for both. The contours surrounding the point *M*=(*κ*_*x*_,*κ*_*y*_)=(3.1113,3.1113) in the Brillouin zone in both figure 13*a*,*b* indicate that *M* is a Dirac-like point. The lowest value *β* contour near *M* in figure 13*a* is *β*=4.37, and in figure 13*b* for the second surface is *β*=4.46. Figure 13*c* shows a magnified picture of the neighbourhood of *M*, for which the *β*=4.40 contour is more visible. We also illustrate an alternative derivation of the *β*=4.40 contours in figure 13*d*, where they take the form of star-like features at the Dirac points. Figure 13*d* also displays the high degree of degeneracy of the light lines at Dirac-like points such as *M*, where we observe multiple intersections. Note that figure 13*b* features the projections of light cone intersections onto the *κ*_*x*_,*κ*_*y*_-plane in the form of lines | |*κ*_*x*_|−|*κ*_*y*_| |=2*π* for the square lattice defined by *η*=1, similar to equation (3.10) in §3b(i).

The dashed line *κ*_*y*_=*κ*_*x*_=3.1113 intersects both the ambient medium's contour $\beta^{2}=\kappa_{x}^{2}+\kappa_{y}^{2}$ and these platonic crystal isofrequency contours *β*=4.40 precisely at the point M. Recalling that the group velocity should be perpendicular to these slowness contours, and in the direction of increasing *β*, we would expect to see refracted and reflected waves at an angle of *π*/4 as illustrated in figure 12*b*.

#### (ix) Interfacial waves for the second dispersion surface

**Figure 14.**
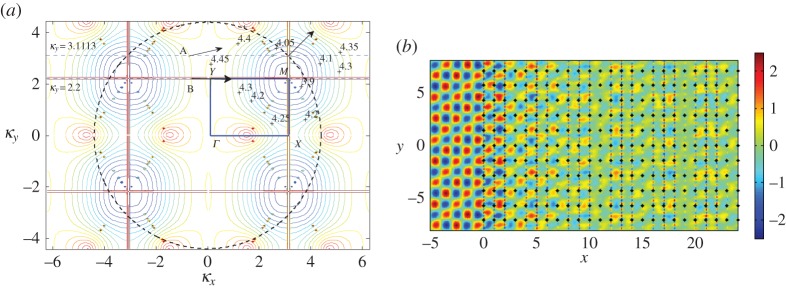
(*a*) Isofrequency curves for the second surface for a rectangular lattice with *d*_*x*_=1, $d_{y}=\sqrt{2}$. The angle of incidence *ψ*=*π*/4, the dashed line *κ*_*y*_=3.1113 and the ambient medium's contour $\beta^{2}=4.4^{2}=\kappa_{x}^{2}+\kappa_{y}^{2}$ are also shown. The predicted directions of propagation for scattered waves are shown by the arrows at A and B for *ψ*=0,*β*=4.40,*κ*_*y*_=3.1113 and *ψ*=*π*/6,*β*=4.45,*κ*_*y*_=2.225, respectively. (*b*) Real part of the displacement field for *β*=4.40, *ψ*=*π*/4, *κ*_*y*_=3.1113 for the rectangular lattice. The Foldy method was used for 2000 gratings. (Online version in colour.)

Now we change the aspect ratio to $\eta =\sqrt{2}$ to obtain a rectangular lattice as in §3b(iii), but keeping all other parameter values the same. In figure 14*a*, we plot the isofrequency contours for the second dispersion surface, ranging from around 3.9 to 4.65. We also add the incident wave arrow for *ψ*=*π*/4 and the dashed line $\kappa_{y}=\beta \sin(\psi )$ = 3.1113. The intersecting line at *κ*_*y*_=3.1113 cuts the *β*=4.40 contour in multiple places, meaning that it is more difficult to predict the behaviour of the system, although intersections corresponding to the group velocity being directed towards the interface from the crystal can be ruled out since the only incoming energy is from the incident medium [31].

The intersection at A indicates that the system supports transmission action in the form of refracted waves at an oblique angle less than *π*/4. This transmission action is visible in figure 14*b*, although at an apparently lower intensity than the reflection observed. Another interesting feature of the real part of the displacement field plotted in figure 14*b* is a wave that appears to be localized within the first three gratings of the half-plane discrete system of pins, which can also be considered as a superposition of several evanescent waves that interfere constructively in the neighbourhood of the structured interface. There is also evidence of coupling between the reflection pattern in the homogeneous plate and the interfacial mode.

**Figure 15.**
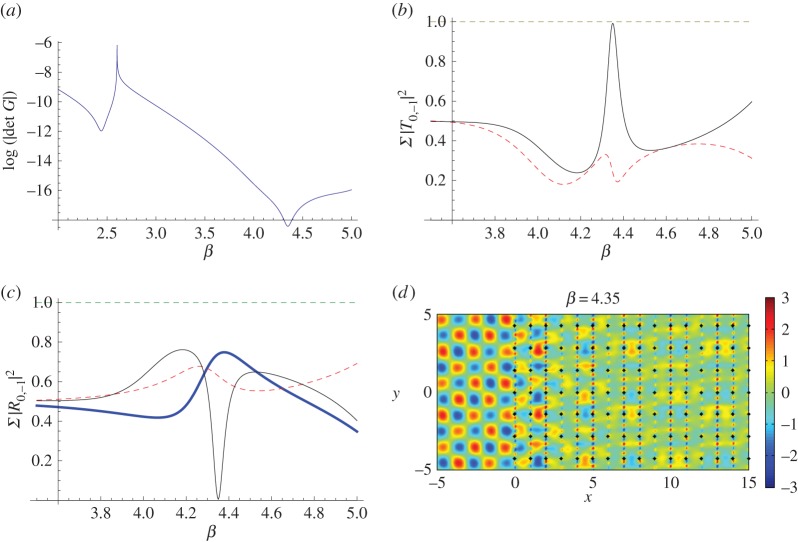
(*a*) Solutions of the eigenvalue problem for a system of three gratings with $d_{y}=\sqrt{2}$ and separation *d*_*x*_=1 for $\kappa_{y}=\beta \sin(\pi /4)$. (*b*) Normalized transmitted energy versus *β* for the triplet's −1 diffraction order (thin solid curve) and the triplet's 0 order (dashed curve) for *ψ*=*π*/4. (*c*) Normalized reflected energy versus *β* for the triplet's −1 diffraction order (thin solid curve) and the first pair's 0 (dashed curve) and −1 (thick solid) orders for *ψ*=*π*/4. (*d*) Real part of displacement for *β*=4.35, *ψ*=*π*/4, *κ*_*y*_=3.0759. The Foldy method was used for 2000 gratings. (Online version in colour.)

It appears that this mode is odd, so that in effect the central grating of the triplet of pinned gratings is not seen, and this demonstrates again the connection with finite-grating stacks discussed in §3b(iii), although here we have coupling between the diffraction orders 0 and −1. We explain this coupling using figure 15, where we show the solutions to the eigenvalue problem for a set of three gratings with period $d_{y}=\sqrt{2}$, separation *d*_*x*_=1.0 and $\kappa_{y}=\beta \sin(\pi /4)$.

Referring to figure 15*a*, the first solution at *β*≈2.5 is due to the zeroth order only, whereas the solution at *β*≈4.35 arises for a mixture of 0 and −1 orders. This value of *β* is very close to *β*=4.40; this explains the localization we observe in figure 14*b*, an effect we optimize by setting the frequency of the system with *β*=4.35.

We show transmitted and reflected energy profiles for the corresponding transmission problem for the first two and three gratings in figure 15*b*,*c*. In figure 15*b*, the solid curve denotes the transmitted energy due to the −1 order for the triplet, explaining how the localized mode helps to support transmission through the rest of the half-plane system. The dashed curve in figure 15*b* represents the transmitted energy for the 0 order, emphasizing that the −1 order dominates. This means that the first pair has to be able to facilitate transmission of the −1 order to reach the third grating. This is illustrated in figure 15*c*, where reflected energy is plotted versus *β*. The thick solid curve shows that the reflection due to the pair's −1 order is around 75%, meaning that there is at least 25% transmission to feed the third grating, which in turn helps to feed the rest of the system.

Figure 15*a*–*c* shows that the maxima for the triplets arise for *β*=4.35 rather than *β*=4.40. Therefore in figure 15*d*, we plot the real part of the displacement field for the same rectangular lattice for *ψ*=*π*/4, but with *β*=4.35 and the corresponding value of *κ*_*y*_ to support Bloch waves along the vertical gratings. The results are similar to those observed in figure 14*b* except that the transmission pattern within the pinned system is slightly more intense than that observed for *β*=4.40.

**Figure 16.**
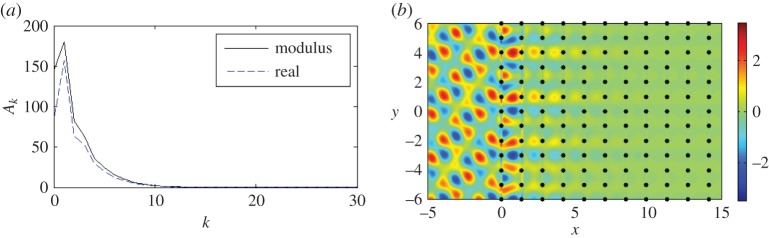
(*a*) Moduli (solid line) and real parts (dashed) of the coefficients *A*_*k*_ for 4000 gratings with *β*=4.45, *ψ*=*π*/6, *κ*_*y*_=2.225. The first 30 of 4000 gratings with $d_{x}=\sqrt{2}$, *d*_*y*_=1.0 are shown. (*b*) Real part of the total displacement field. (Online version in colour.)

A similar but more intense effect is observed for *β*=4.45 at *Y* with *ψ*=*π*/6 and *κ*_*y*_=2.225. The resultant propagation parallel to *κ*_*y*_=0 is illustrative of the uni-directional localized modes associated with such parabolic profiles, and can be rotated through *π*/2 by swapping the horizontal and vertical periods of the lattice. Thus for a lattice of 4000 gratings with period *d*_*y*_=1.0 and spacing $d_{x}=\sqrt{2}$, we show an interfacial wave in figure 16*b* propagating in the vertical direction parallel to *κ*_*x*_=0, as predicted by the corresponding isofrequency contour, and very little wave action inside the pins, as predicted by the decay of the coefficients in figure 16*a*.

#### (x) Higher frequencies—interfacial waves

**Figure 17.**
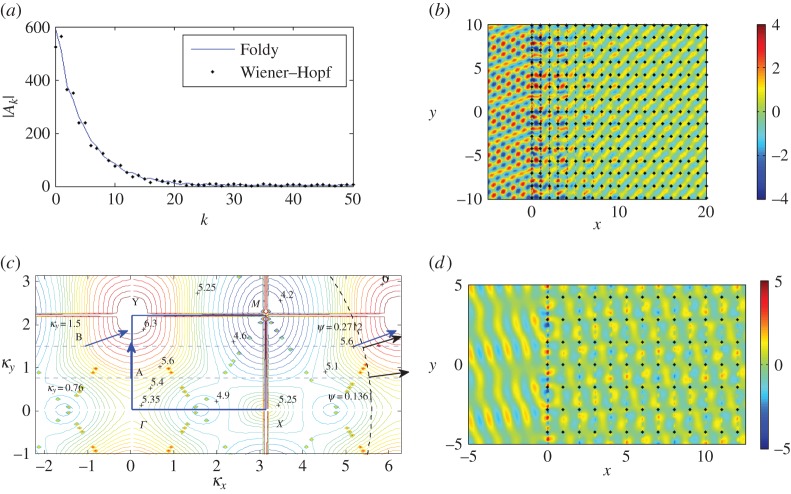
A plane wave is incident at *ψ*=0.1361 on a lattice of 2000 gratings with *d*_*x*_=1.0, $d_{y}=\sqrt{2}$ for *β*=5.6, *κ*_*y*_=0.76. (*a*) Moduli of the coefficients |*A*_*k*_| with the first 50 shown for both Foldy (solid curve) and Wiener–Hopf (dots). (*b*) Real part of the total displacement field. (*c*) Magnification of the third dispersion surface. (*d*) Real part of the total displacement field for *β*=5.6, *ψ*=0.2712, *κ*_*y*_=1.5. (Online version in colour.)

It is clear that, as the frequency *ω*=*β*^2^ increases, a more complicated picture emerges. We illustrate this with an example for *β*=5.6 in figure 17*a*,*b*. The third surface illustrated in figure 6 and magnified here in figure 17*c* possesses many important features. At the point *Γ*, the contours change direction by *π*/2 for *β*≈5.365 and there is a Dirac-like point at *X* for *β*≈5.45. This narrow frequency window supports an array of interesting wave phenomena, similar to those discussed for the first surface in §3b(iii).

In figure 17*c*, we show a collection of isofrequency contours for the platonic crystal's third dispersion surface, as well as the contour for the homogeneous biharmonic plate medium—an arc of the dashed circle defined by $\beta^{2}=\kappa_{x}^{2}+\kappa_{y}^{2}$ for the case *β*=5.6. We also refer again to equation (3.10) to explain the appearance of the light cone projections on the isofrequency diagram.

We seek an interfacial wave so we choose *ψ* such that $\kappa_{y}=\beta \sin(\psi )$ is tangent to the *β*=5.6 contour for the third surface (point A in figure 17*c*). The vertical arrow predicts the propagation direction of the refracted waves for this *κ*_*y*_=0.76 and corresponding *ψ*=0.1361. In this way, we predict that some refracted waves will propagate perpendicularly to this contour, i.e. in a vertical direction parallel to the interface. We plot the real part of the displacement field for these parameter settings in figure 17*b*, along with the moduli for the coefficients |*A*_*k*_| in figure 17*a* generated by both the Foldy and Wiener–Hopf methods.

Figure 17*b* shows low propagation within the pinned structure, and relatively low reflection, compared with figure 15*b* for example. However, there is clearly a travelling wave localized within the first few gratings, albeit with a relatively long wavelength. The exponential decay of the moduli of the scattering coefficients |*A*_*k*_| is illustrated in figure 17*a*. In figure 17*d*, we consider an example for an arbitrary value of *κ*_*y*_ to highlight the contrast in behaviour for various *ψ* and *κ*_*y*_. For *κ*_*y*_=1.5 and *ψ*=0.2712, we show the real part of the total displacement field in figure 17*d*. Although there is increased intensity at the interface, as one would expect, the dominant behaviour is propagation and in almost the same direction as the incident wave. This is predicted by the direction of the arrows at B and on the *β*=5.6 contour close to the incident wave's arrow *ψ*=0.2712 in figure 17*c*.

Finally, we consider the case *ψ*=0, *κ*_*y*_=0. At the point *Γ* in figure 17*c*, the contours for *β*=5.35 support propagation in a direction normal to those of *β*=5.40, which support propagation parallel to the interface similar to A for *β*=5.6 for *ψ*=0.1361 in figure 17*b*,*c*. Thus, there is a point of inflection for 5.35<*β*<5.40 and it occurs for *β*≈5.365. For *β*≤5.365, the refracted waves propagate through the pinned system rather than along its edge, which is what happens for 5.365<*β*<5.45. However at the Dirac-like point *X*; *β*≈5.45, the waves exhibit a mixture of edge localization and propagation through the system. This is because we have the third, fourth and fifth dispersion surfaces meeting at this frequency, leading to a combination of behaviours. The third surface (figure 17*c*) predicts propagation along the edge for *β*=5.45 contours while the fourth and fifth surfaces predict propagation parallel to *κ*_*y*_=0, into the pinned lattice.

**Figure 18.**
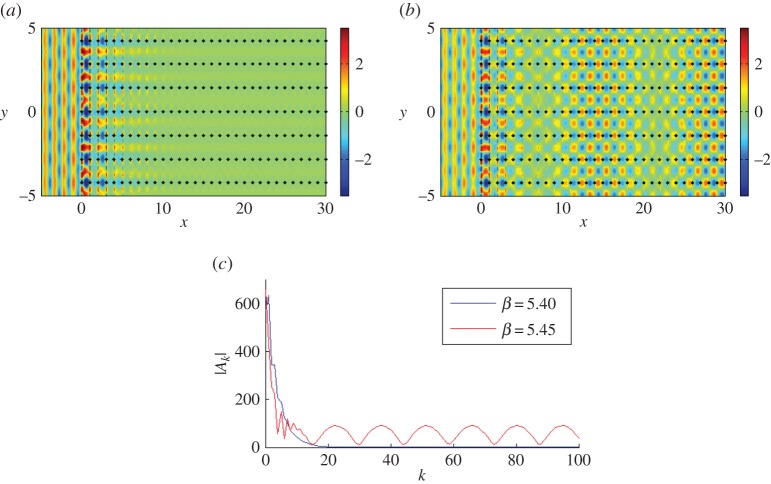
A plane wave is incident at *ψ*=0 on a lattice of 4000 gratings with *d*_*x*_=1.0, $d_{y}=\sqrt{2}$. (*a*,*b*) Real part of the total displacement field for *β*=5.40, *β*=5.45. (*c*) Comparison of the moduli of coefficients |*A*_*k*_|. (Online version in colour.)

We observe these properties in figure 18, where parts figure 18*a*,*b* shows the real part of the total displacement field for *β*=5.40 and 5.45, respectively. The amplitudes are extraordinarily large for *β*=5.365, where the isofrequency contours are about to change direction to support interfacial waves rather than propagation through the pinned system (see *Γ* in figure 17*c*). This transition has occurred in figure 18*a* for *β*=5.40, where the localized interface mode clearly dominates any action inside the pinned region. The comparison of the coefficients *A*_*k*_ in figure 18*c* also demonstrates the decay of the coefficients to zero inside the crystal for *β*=5.40. However *β*=5.45 close to a Dirac-like point supports both edge localization and wave propagation as illustrated in figure 18*b*,*c*, where a wave is seen to propagate through the pinned lattice with the wavelength of the envelope function matching that of the coefficients.

## 4. Concluding remarks: interfacial waves and dynamic localization

The theory and examples presented in this paper have identified novel regimes, typical of flexural waves in structured Kirchhoff–Love plates, for the case of a semi-infinite structured array of rigid pins in an otherwise homogeneous plate. The interface waveguide modes have been identified and studied here.

For a half-plane occupied by periodically distributed rigid pins, we have demonstrated an interplay between the transmission/reflection properties at the interface and dispersion properties of Floquet–Bloch waves in an infinite doubly periodic constrained plate. Specifically, we have analysed regimes corresponding to frequencies and wavevectors that determine stationary points on the dispersion surfaces, as well as Dirac-like points. For such regimes, we have demonstrated that the structure supports localized interfacial waves, among other dynamic effects.

The localization was predicted by an analytical solution, and a formal connection has been shown here between the doubly quasi-periodic Green's function for an infinite plane and the system of equations required to obtain the intensities of sources at the rigid pins, which occupy the half-plane.

## Supplementary Material

Supplementary material

## Data accessibility

The work does not include any experimental data. All computational results presented in the examples of the paper are reproducible using the analytical methods detailed in the article and the appendices of the electronic supplementary material.

## References

[1] Pendry JB 2000 Negative refraction makes a perfect lens. *Phys. Rev. Lett.* 85, 3966–3969. (doi:10.1103/PhysRevLett.85.3966) 11041972 10.1103/PhysRevLett.85.3966

[2] McPhedran RC, Movchan AB, Movchan NV 2009 Platonic crystals: Bloch bands, neutrality and defects. *Mech. Mater.* 41, 356–363. (doi:10.1016/j.mechmat.2009.01.005)

[3] Farhat M, Guenneau S, Enoch S 2010 High-directivity and confinement of flexural waves through ultrarefraction in thin perforated plates. *Eur. Phys. Lett.* 91, 54003 (doi:10.1209/0295-5075/91/54003)

[4] Farhat M, Guenneau S, Enoch S, Movchan AB, Petursson G 2010 Focussing bending waves via negative refraction in perforated thin plates. *Appl. Phys. Lett.* 96, 081909 (doi:10.1063/1.3327813)

[5] Dubois M, Farhat M, Bossy E, Enoch S, Guenneau S, Sebbah P 2013 Flat lens for pulse focusing of elastic waves in thin plates. *Appl. Phys. Lett.* 103, 071915 (doi:10.1063/1.4818716)

[6] Antonakakis T, Craster RV, Guenneau S 2013 Moulding flexural waves in elastic plates lying atop a Faqir's bed of nails. (http://arxiv.org/abs/1301.7653) 10.1098/rspa.2012.0533 PMC3637003 23633908

[7] Torrent D, Mayou D, Sánchez-Dehesa J 2013 Elastic analog of graphene: Dirac cones and edge states for flexural waves in thin plates. *Phys. Rev. B* 87, 115143 (doi:10.1103/PhysRevB.87.115143)

[8] McPhedran RC, Movchan AB, Movchan NV, Brun M, Smith MJA 2015 ‘Parabolic’ trapped modes and steered Dirac cones in platonic crystals. *Proc. R. Soc. A* 471, 20140746 (doi:10.1098/rspa.2014.0746) 10.1098/rspa.2014.0746 PMC4984980 27547089

[9] Mace BR 1980 Periodically stiffened fluid-loaded plates, I: response to convected harmonic pressure and free wave propagation. *J. Sound Vib.* 73, 473–486. (doi:10.1016/0022-460X(80)90662-8)

[10] Mead DJ 1996 Wave propagation in continuous periodic structures: research contributions from Southampton 1964–1995. *J. Sound Vib.* 190, 495–524. (doi:10.1006/jsvi.1996.0076)

[11] Movchan AB, Movchan NV, McPhedran RC 2007 Bloch–Floquet bending waves in perforated thin plates. *Proc. R. Soc. A* 463, 2505–2518. (doi:10.1098/rspa.2007.1886)

[12] Antonakakis T, Craster RV 2012 High frequency asymptotics for microstructured thin elastic plates and platonics. *Proc. R. Soc. Lond. A* 468, 20110652 (doi:10.1098/rspa.2011.0652)

[13] Evans DV, Porter R 2007 Penetration of flexural waves through a periodically constrained thin elastic plate floating in *vacuo* and floating on water. *J. Eng. Math.* 58, 317–337. (doi:10.1007/s10665-006-9128-0)

[14] Movchan NV, McPhedran RC, Movchan AB, Poulton C 2009 Wave scattering by platonic grating stacks. *Proc. R. Soc. A* 465, 3383–3400. (doi:10.1098/rspa.2009.0301)

[15] Poulton CG, McPhedran RC, Movchan NV, Movchan AB 2010 Convergence properties and flat bands in platonic crystal band structures using the multipole formulation. *Wave Random Complex Media* 20, 702–716. (doi:10.1080/17455030903203140)

[16] Haslinger SG, Movchan NV, Movchan AB, McPhedran RC 2012 Transmission, trapping and filtering of waves in periodically constrained elastic plates. *Proc. R. Soc. A* 468, 20110318 (doi:10.1098/rspa.2011.0318)

[17] Wiener N, Hopf E 1931 Über eine Klasse singulärer Integralgleichungen. *Sitz. Berlin Akad. Wiss* 31, 696–706.

[18] Noble B 1958 *Methods based on the Wiener–Hopf technique for the solution of partial differential equations*. London, UK: Pergamon Press.

[19] Fel'd AN 1955 An infinite system of linear algebraic equations connected with the problem of a semi-infinite periodic structure. *Dokl. Akad. Nauk SSSR* 102, 257–260.

[20] Slepyan LI 2002 *Models and phenomena in fracture mechanics*. Berlin, Germany: Springer.

[21] Wasylkiwskyj W 1973 Mutual coupling effects in semi-infinite arrays. *IEEE Trans. Ant. Prop.* 21, 277–285. (doi:10.1109/TAP.1973.1140507)

[22] Linton CM, Martin PA 2004 Semi-infinite arrays of isotropic point scatterers. A unified approach. *SIAM J. Appl. Math.* 64, 1035–1056. (doi:10.1137/S0036139903427891)

[23] Tymis N, Thompson I 2011 Low frequency scattering by a semi-infinite lattice of cylinders. *Q. J. Mech. Appl. Math.* 64, 171–195. (doi:10.1093/qjmam/hbr001)

[24] Hills NL, Karp SN 1965 Semi-infinite diffraction gratings I. *Commun. Pure Appl. Math.* 18, 203–233. (doi:10.1002/cpa.3160180119)

[25] Karp S 1952 Diffraction by finite and infinite gratings. *Phys. Rev.* 86, 586.

[26] Abramowitz M, Stegun IA 1964 *Handbook of mathematical functions*. Washington, DC: National Bureau of Standards.

[27] Haslinger SG, Movchan AB, Movchan NV, McPhedran RC 2014 Symmetry and resonant modes in platonic grating stacks. *Waves Random Complex Media* 24, 126–148. (doi:10.1080/17455030.2014.884733)

[28] Foldy LL 1945 The multiple scattering of waves I. General theory of isotropic scattering by randomly distributed scatterers. *Phys. Rev.* 67, 107–119. (doi:10.1103/PhysRev.67.107)

[29] Born M, Wolf E 1959 *Principles of optics*. London, UK: Pergamon Press.

[30] Zengerle R 1987 Light propagation in singly and doubly periodic waveguides. *J. Mod. Opt.* 34, 1589–1617. (doi:10.1080/09500348714551531)

[31] Joannopoulos JD, Johnson SG, Winn JN, Meade RD 2008 *Photonic crystals: molding the flow of light*, 2nd edn Princeton, NJ: Princeton University Press.

[32] Farhat M, Chen P-Y, Bagci H, Enoch S, Guenneau S, Alù A 2014 Platonic scattering cancellation for bending waves in a thin plate. *Nat. Sci. Rep.* 4, 4644 (doi:10.1038/srep04644) 10.1038/srep04644 PMC4027886 24844801

