# Supplementary material for: Dynamic interfacial trapping of flexural waves in structured plates
Source: Proc Math Phys Eng Sci. 2016 Feb;472(2186):20150658. doi: 10.1098/rspa.2015.0658 (PMC4841657; doi:10.1098/rspa.2015.0658)
Supplement: Supplementary material [file rspa20150658supp1.zip › rspa-2015-0658-File007/source_files_suppmat_sgh/PROCEEDINGS_A_RGB.PDF]

PROCEEDINGS A
