# Supplementary material for: Dynamic interfacial trapping of flexural waves in structured plates
Source: Proc Math Phys Eng Sci. 2016 Feb;472(2186):20150658. doi: 10.1098/rspa.2015.0658 (PMC4841657; doi:10.1098/rspa.2015.0658)
Supplement: Supplementary material [file rspa20150658supp1.zip › rspa-2015-0658-File007/source_files_suppmat_sgh/Semi_Infinite_gratings_suppmat_Jan_21.pdf]

PROCEEDINGS A

rspa.royalsocietypublishing.org

Research

Article submitted to journal

Subject Areas:
Applied Mathematics

Keywords:
Flexural waves, platonic crystals, discrete Wiener-Hopf method, localisation and neutrality.

Author for correspondence:
S.G. Haslinger

Dynamic interfacial trapping of flexural waves in structured plates - Supplementary Material

S. G. Haslinger [1], R. V. Craster [2], A. B. Movchan [1], N. V. Movchan [1] and I. S. Jones [3]

[1] Department of Mathematical Sciences, Mathematical Sciences Building, University of Liverpool, Peach Street, Liverpool L69 7ZL, United Kingdom
[2] Department of Mathematics, Imperial College London, London SW7 2AZ, UK
[3] School of Engineering, Liverpool John Moores University, Liverpool L3 3AF, UK

THE ROYAL SOCIETY PUBLISHING

© The Author(s) Published by the Royal Society. All rights reserved.

A. Factorizing $\mathcal{K}(z)$

A critical technical detail is the factorization $\mathcal{K}(z) = \mathcal{K}_+(z)\mathcal{K}_-(z)$ with $\mathcal{K}_+, \mathcal{K}_-$ respectively analytic inside and outside the unit circle. Laurent's theorem gives

$$\log \mathcal{K}(z) = \frac{1}{2\pi i}\int_{C_+} \frac{\log \mathcal{K}(\varrho)}{\varrho - z} d\varrho - \frac{1}{2\pi i}\int_{C_-} \frac{\log \mathcal{K}(\varrho)}{\varrho - z} d\varrho, \tag{A.1}$$

where C_+ is a circle of radius c_+ slightly larger than the unit circle, and C_- is a circle of radius c_- slightly smaller than the unit circle (see figure 2).The upper bound for C_+ is γ_+, and the lower bound for C_- is γ_-. The desired factorization is

$$\mathcal{K}(z) = \exp\left\{\frac{1}{2\pi i}\int_{C_+} \frac{\log \mathcal{K}(\varrho)}{\varrho - z} d\varrho\right\} \exp\left\{\frac{-1}{2\pi i}\int_{C_-} \frac{\log \mathcal{K}(\varrho)}{\varrho - z} d\varrho\right\} = \mathcal{K}_+(z)\mathcal{K}_-(z). \tag{A.2}$$

We note here that ϱ is of the form $e^{i(\theta \pm i\delta)}$ for $C_\mp$, with $z = e^{i\theta}$. It is also assumed that $C_\pm$ are chosen so that $\log \mathcal{K}$ is well defined.

Referring to the factorization (A.2),

$$\mathcal{K}_\pm(z) = \exp\left\{\frac{\pm 1}{2\pi i}\int_{C_\pm} \frac{\log \mathcal{K}(\varrho)}{\varrho - z} d\varrho\right\}$$

is evaluated for $z = e^{i\theta_1}$ for some argument θ_1 on the unit circle, with $\varrho = c_\pm e^{i\theta}$ for $0 \le \theta \le 2\pi$. Here we outline our chosen method for the second step of regularisation (the first step being the determination of $\gamma_\pm$ using $\beta_\epsilon = \beta + i\epsilon$). Defining $\varrho = \varrho_\pm e^{i\theta}$ with $\varrho_\pm = \exp\{\pm\delta\}$, we have

$$d\varrho = i\varrho_\pm e^{i\theta} d\theta$$

and

$$\mathcal{K}_\pm(z) = \exp\left\{\frac{\pm 1}{2\pi}\int_0^{2\pi} \frac{\log \mathcal{K}(\varrho_\pm e^{i\theta})\varrho_\pm e^{i\theta}}{\varrho_\pm e^{i\theta} - z} d\theta\right\}.$$

Then we may write

$$\mathcal{K}_\pm(z) = \exp\left\{\frac{\pm 1}{2\pi}\int_0^{2\pi} \frac{\log \mathcal{K}(\varrho_\pm e^{i\theta})}{1 - e^{\mp\delta}e^{i(\theta_1-\theta)}} d\theta\right\} = \exp\left\{\frac{\pm\varrho_\pm}{2\pi}\int_0^{2\pi} \frac{\log \mathcal{K}(\varrho_\pm e^{i\theta})}{\varrho_\pm - e^{i(\theta_1-\theta)}} d\theta\right\}. \tag{A.3}$$

The regularisation method involving $\beta_\epsilon = \beta + i\epsilon$ and $\varrho = \varrho_\pm e^{i\theta}$ with $\varrho_\pm = \exp\{\pm\delta\}$ achieves good agreement with the Foldy computations for the truncated semi-infinite gratings.

B. Accelerated convergence for $\mathcal{K}(z)$

The biharmonic operator's kernel $\mathcal{K}(z)$ converges extremely slowly because of the highly oscillatory nature of the Hankel function terms. The direct correspondence with the quasi-periodic grating Green's function for a specific definition of $z = \exp\{i\kappa_x s\}$, where s denotes horizontal spacing, enables us to implement some known accelerated convergence techniques. Twersky (1961) investigated the convergence of the Schlömilch series

$$\sum_{p=1}^{\infty} Z_{2l}(pD)\cos(pD\sin\psi_0), \tag{B.1}$$

$$\sum_{p=1}^{\infty} Z_{2l+1}(pD)\sin(pD\sin\psi_0), \tag{B.2}$$

where Z_n is the nth order Bessel function and $D > 0$, $0 \le \sin\psi_0 < 1$. In particular, we consider the series in the form

$$\mathcal{H}_n = \sum_{p=1}^{\infty} H_n^{(1)}(pD)\,[\exp\{ip\,D\sin\psi_0\}(-1)^n + \exp\{-ip\,D\sin\psi_0\}]. \tag{B.3}$$

Here $H_n^{(1)} = J_n + iN_n$ is the standard Hankel function of the first kind, with J_n and N_n being the Bessel and Neumann functions. It is clear that D can be replaced by βs to coincide with our treatment, and we note that $\beta s\sin(\psi_0)$ is associated with the Bloch parameter κ_x for the periodic structure we are considering.

It is well-known that the representation (B.3) is too slowly convergent for practical use. Twersky (1961) derived an alternative rapidly convergent representation in terms of elementary functions. However he did not evaluate the $n = 0$ case which we require here, but instead referred to the work of Magnus & Oberhettinger (1948). Rewriting the representation for the kernel function (2.9) in the form

$$\mathcal{K}(z) = \frac{i}{8\beta^2}\sum_{j=1}^{\infty}\left[H_0^{(1)}(\beta sj) + \frac{2i}{\pi}K_0(\beta sj)\right](z^j + z^{-j}) + \frac{i}{8\beta^2}, \tag{B.4}$$

we substitute $n = 0$ in (B.3) to obtain the series

$$2\sum_{p=1}^{\infty} H_0^{(1)}(pD)\,\cos(p\,D\sin\psi_0), \tag{B.5}$$

which is immediately associated with the slowly convergent part of representation (B.4) for z on the unit circle, with $D = \beta s$ and $z = e^{i\theta}$, $\theta = \beta s\sin\psi_0 = \kappa_x s$. For z sitting precisely on the unit circle, we may use the accelerated convergence formulae of Movchan *et al.* (2009) which involve grating sums S_0^H for the Hankel functions, and S_0^K for the Bessel K functions:

$$S_0^H + \frac{2i}{\pi}S_0^K + 1 = \frac{2}{s}\left[\sum_p\left(\frac{1}{\chi_p} - \frac{1}{\hat{\chi}_p}\right) + i\sum_p\left(\frac{1}{|\hat{\chi}_p|} - \frac{1}{\hat{\chi}_p}\right)\right], \tag{B.6}$$

where the right-hand sum is made up of propagating and evanescent parts, and is cubically convergent. The terms χ_p and $\hat{\chi}_p = i\tau_p,\ \tau_p > 0$ are defined by

$$\kappa_p = \kappa_x + \frac{2\pi p}{s}, \tag{B.7}$$

$$\chi_p = \begin{cases}\sqrt{\beta^2 - {\kappa_p}^2} & ,\ {\kappa_p}^2 \le \beta^2,\\ i\sqrt{{\kappa_p}^2 - \beta^2} & ,\ {\kappa_p}^2 > \beta^2,\end{cases} \tag{B.8}$$

$$\tau_p = \sqrt{\beta^2 + \kappa_p^2}, \tag{B.9}$$

where $p \in \mathbb{Z}$. There is a finite number of propagating orders p (the $1/\chi_p$ terms in (B.6)) with all other orders being evanescent.

Branch cuts arise from the Helmholtz part of the kernel function $\mathcal{K}(z)$ (2.9) and not from the modified Helmholtz part. This is illustrated in figure B1, where part (a) shows the contribution from the sum of Hankel functions, and part (b) shows the real part of the Bessel K contributions (the imaginary part is of order 10^{-20}). Not only does figure B1(b) illustrate the exponentially small contribution from the modified Helmholtz operator, but also that it is a well-behaved function with no branch cuts. These branch cuts arise from the grating sums which include a factor of the form $1/(\beta^2 - (\kappa_x + 2\pi p/s)^2)$, which is often used to determine the "light lines" for the system.

(a) Series representations for $\mathcal{K}(z)$ for numerical evaluation

The accelerated convergence formulae of equation (B.6) are only valid for $\mathcal{K}(z)$ if z lies on the unit circle. Expressions for $\mathcal{K}_+(z)$ and $\mathcal{K}_-(z)$ in (A.2) involve ϱ which lies either just inside or outside the unit circle. Therefore to evaluate $\mathcal{K}_+$ and $\mathcal{K}_-$ we must use the direct form for $\mathcal{K}(z)$ (B.4)

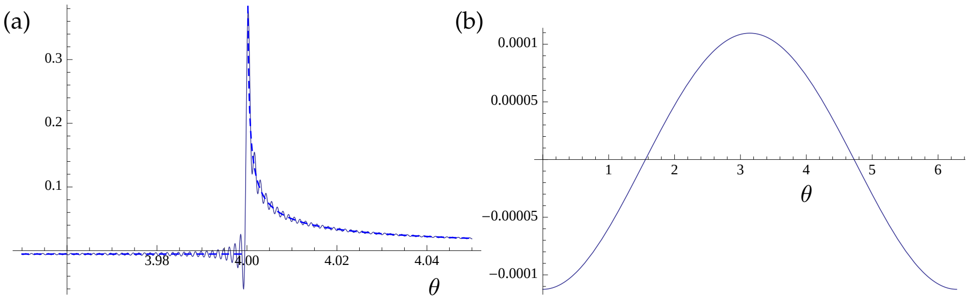

Figure B1: (a) Imaginary part of the Helmholtz part of the kernel function for $\beta = 4.0$, $s = 1.0$ with $z = e^{i\theta}$ for $\theta \in [3.95, 4.05]$ using 5000 terms (solid line) and convergent grating sums (dashed line). (b) Real part of the modified Helmholtz part of the kernel function for the same parameter values with 5000 terms.

with either regularisation or the use of a remainder function which employs a finite number of terms directly and adds an infinite tail evaluated using asymptotic approximations. For the latter treatment, we rewrite $\mathcal{K}(z)$ in the form

$$\mathcal{K}(z) = \frac{i}{8\beta^2} \sum_{j=N+1}^{\infty} \left[H_0^{(1)}(\beta s j) + \frac{2i}{\pi} K_0(\beta s j) \right] (z^j + z^{-j}) + \frac{i}{8\beta^2} \sum_{j=1}^{N} \left[H_0^{(1)}(\beta s j) + \frac{2i}{\pi} K_0(\beta s j) \right] (z^j + z^{-j}) + \frac{i}{8\beta^2}, \tag{B.10}$$

where N denotes a finite number of terms for direct application of the kernel series, with the remainder evaluated via a function we define as $R(z)$ based on the asymptotic analysis by HK. We write

$$R(z) = \sum_{j=N+1}^{\infty} H_0^{(1)}(\beta s j)(z^j + z^{-j}) = \sum_{n=1}^{\infty} H_0^{(1)}(\beta s(n+N))(z^{n+N} + z^{-(n+N)}), \tag{B.11}$$

where we have used the change of index of summation $n = j - N$.

We replace the Hankel functions by their asymptotic forms for large βs, since we are only considering the tail of $\mathcal{K}(z)$. Hence,

$$R(z) = e^{-i\pi/4} \sqrt{\frac{2}{\beta s}} \sum_{n=1}^{\infty} \frac{(z e^{i\beta s})^{n+N}}{\sqrt{\pi(n+N)}} + e^{-i\pi/4} \sqrt{\frac{2}{\beta s}} \sum_{n=1}^{\infty} \frac{\left(\frac{1}{z} e^{i\beta s}\right)^{n+N}}{\sqrt{\pi(n+N)}}, \tag{B.12}$$

for which we define the function $F(z)$ by

$$F(z) = \sum_{n=1}^{\infty} \frac{z^{n+N}}{\sqrt{\pi(n+N)}} = \frac{2}{\pi} \sum_{n=1}^{\infty} \int_0^{\infty} z^{n+N} e^{-t^2(n+N)} dt, \tag{B.13}$$

where we refer to Appendix 1 of HK. For $|z| < 1$, we may interchange the order of summation and integration such that

$$F(z) = \frac{2}{\pi} \int_0^{\infty} \sum_{n=1}^{\infty} b^{n+N} dt, \quad b = z e^{-t^2}, \quad |b| < 1. \tag{B.14}$$

Thus,

$$F(z) = \frac{2}{\pi} \int_0^{\infty} \frac{b^{N+1}\, dt}{1 - b}$$

and

$$R(z) = e^{-i\pi/4}\sqrt{\frac{2}{\beta s}}\left\{F(ze^{i\beta s}) + F\left(\frac{1}{z}e^{i\beta s}\right)\right\}, \quad F(z) = \frac{2}{\pi}z^{N+1}\int_0^\infty \frac{e^{-t^2(N+1)}\,dt}{1 - ze^{-t^2}}. \tag{B.15}$$

Thus, referring to equation (B.10) we obtain

$$\mathcal{K}(z) = \frac{i}{8\beta^2}\left(\sum_{j=1}^{N}\left[H_0^{(1)}(\beta s j) + \frac{2i}{\pi}K_0(\beta s j)\right](z^j + z^{-j}) + R(z) + 1\right). \tag{B.16}$$

This more amenable representation for $\mathcal{K}(z)$ enables us to determine explicit expressions for $\mathcal{K}_+(z)$ and $\mathcal{K}_-(z)$ of equation (A.2) which are required to evaluate $A_+(z)$ (2.19). However with regularisation, the introduction of $\beta_\epsilon = \beta + i\epsilon$ ensures that the sums converge sufficiently quickly to avoid the necessity of using the remainder function $R(z)$ for $\mathcal{K}_+(z)$ and $\mathcal{K}_-(z)$ away from the unit circle.
