# Supplementary figures and images for: Dynamic interfacial trapping of flexural waves in structured plates
Source: Proc Math Phys Eng Sci. 2016 Feb;472(2186):20150658. doi: 10.1098/rspa.2015.0658 (PMC4841657; doi:10.1098/rspa.2015.0658)

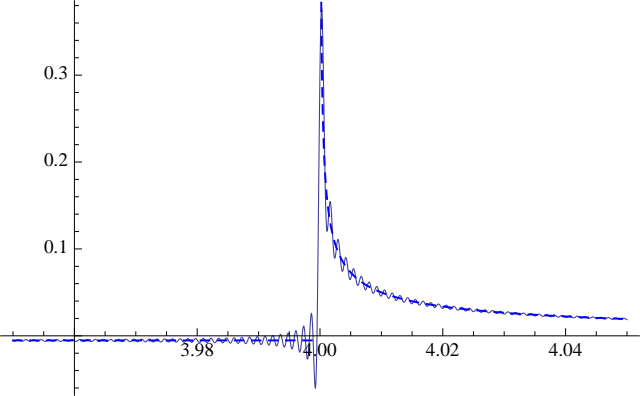

Supplement: Supplementary material [file rspa20150658supp1.zip › rspa-2015-0658-File007/source_files_suppmat_sgh/branchcuts1.pdf]

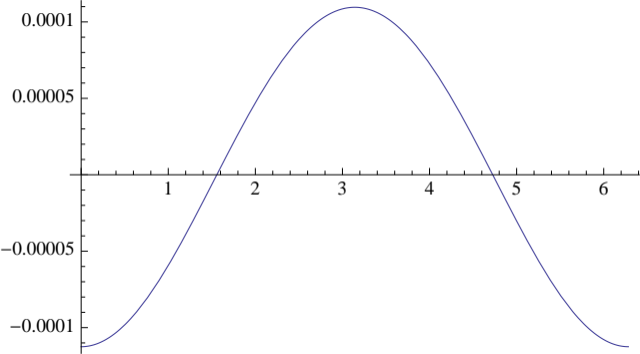

Supplement: Supplementary material [file rspa20150658supp1.zip › rspa-2015-0658-File007/source_files_suppmat_sgh/real_nobranchcut_K_beta4.pdf]

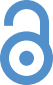

Supplement: Supplementary material [file rspa20150658supp1.zip › rspa-2015-0658-File007/source_files_suppmat_sgh/RSTA_OpenAccesslogo_RGB.PDF]

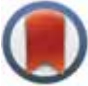

CrossMark

[click for updates](#)

Supplement: Supplementary material [file rspa20150658supp1.zip › rspa-2015-0658-File007/source_files_suppmat_sgh/RS_crossmark_logo.pdf]
